# Unintended Consequences of mHealth Interactive Voice Messages Promoting Contraceptive Use After Menstrual Regulation in Bangladesh: Intimate Partner Violence Results From a Randomized Controlled Trial

**DOI:** 10.9745/GHSP-D-19-00015

**Published:** 2019-09-23

**Authors:** Kate Reiss, Kathryn Andersen, Erin Pearson, Kamal Biswas, Fahmida Taleb, Thoai D. Ngo, Altaf Hossain, Sharmani Barnard, Chris Smith, James Carpenter, Jamie Menzel, Katharine Footman, Katherine Keenan, Megan Douthwaite, Yasmin Reena, Hassan Rushekh Mahmood, Tanzila Tabbassum, Manuela Colombini, Loraine Bacchus, Kathryn Church

**Affiliations:** a Department of Population Health, London School of Hygiene and Tropical Medicine, London, UK.; b Ipas, Chapel Hill, NC, USA.; c Ipas Bangladesh, Dhaka, Bangladesh.; d Marie Stopes Bangladesh, Dhaka, Bangladesh.; e Population Council, New York, NY, USA.; f Association for Prevention of Septic Abortion, Bangladesh, Dhaka, Bangladesh.; g School of Population Health and Environmental Sciences, King's College, London, UK.; h School of Tropical Medicine and Global Health, Nagasaki University, Nagasaki, Japan.; i Department of Medical Statistics, London School of Hygiene and Tropical Medicine, London, UK.; j Marie Stopes International, London, UK.; k School of Geography and Sustainable Development, University of St. Andrews, St. Andrews, UK.; l icddr,b, Dhaka, Bangladesh.; m Department of Global Health and Development, London School of Hygiene and Tropical Medicine, London, UK.

## Abstract

**Background:**

Mobile phones for health (mHealth) hold promise for delivering behavioral interventions. We evaluated the effect of automated interactive voice messages promoting contraceptive use with a focus on long-acting reversible contraceptives (LARCs) among women in Bangladesh who had undergone menstrual regulation (MR), a procedure to “regulate the menstrual cycle when menstruation is absent for a short duration.”

**Methods:**

We recruited MR clients from 41 public- and private-sector clinics immediately after MR. Eligibility criteria included having a personal mobile phone and consenting to receive messages about family planning by phone. We randomized participants remotely to an intervention group that received at least 11 voice messages about contraception over 4 months or to a control group (no messages). The primary outcome was LARC use at 4 months. Adverse events measured included experience of intimate partner violence (IPV). Researchers recruiting participants and 1 analyst were blinded to allocation groups. All analyses were intention to treat. The trial is registered with ClinicalTrials.gov (NCT02579785).

**Results:**

Between December 2015 and March 2016, 485 women were allocated to the intervention group and 484 to the control group. We completed follow-up on 389 intervention and 383 control participants. Forty-eight (12%) participants in the intervention group and 59 (15%) in the control group reported using a LARC method at 4 months (adjusted odds ratio [aOR] using multiple imputation=0.95; 95% confidence interval [CI]=0.49 to 1.83; *P*=.22). Reported physical IPV was higher in the intervention group: 42 (11%) intervention versus 25 (7%) control (aOR=1.97; 95% CI=1.12 to 3.46; *P*=.03) when measured using a closed question naming acts of violence. No violence was reported in response to an open question about effects of being in the study.

**Conclusions:**

The intervention did not increase LARC use but had an unintended consequence of increasing self-reported IPV. Researchers and health program designers should consider possible negative impacts when designing and evaluating mHealth and other reproductive health interventions. IPV must be measured using closed questions naming acts of violence.

## Abstract (precis)

Automated interactive voice messages about post-menstrual regulation contraception delivered to women in Bangladesh via mobile phone were associated with increased reports of intimate partner violence. This finding highlights the importance of taking steps to minimize risk when delivering phone messages on sensitive topics and the need for assessing violence in such situations.

## INTRODUCTION

Bangladesh has seen a rapid increase in use of contraception over the last 40 years; however, among married women in Bangladesh, 12% wanted to delay or stop childbearing but were not using a method in 2014.^1^ Furthermore, 48% of all pregnancies in Bangladesh are estimated to be unintended, leading to health, social, and economic costs for women and their families.^2^^,^^3^ The type of contraceptives women use has implications for unintended pregnancies: long-acting reversible contraceptives (LARCs)—intrauterine devices (IUDs) and implants—are the most effective reversible methods, with failure rates of between 0.1% and 0.8% in the first year of typical use.^4^ In contrast, short-acting methods have higher typical-use failure rates of 4% (the injectable), 7% (the pill), and 13% (male condoms).^4^ LARCs also have low levels of discontinuation and high user satisfaction.^5^^,^^6^ In Bangladesh, 62% of married women of reproductive age use contraception, but just 2% use a LARC and 30% of contraceptive users discontinue their method within a year.^1^ Although awareness of contraceptive methods in Bangladesh is high,^7^ many women lack accurate information on method attributes, particularly for long-acting methods, and fear of negative effects is common.^7^ Increased information provision about LARCs has been shown to increase uptake of these methods.^8^

Women terminating a pregnancy are a key group to reach with information about contraception as they usually want to delay or prevent future pregnancies. Legally, abortion is available in Bangladesh only to save a woman's life,^3^ but menstrual regulation (MR), “the procedure of regulating the menstrual cycle when menstruation is absent for a short duration,”^9^ is authorized in public and government-approved NGO and private health facilities up to 12 weeks since the last menstrual period.^10^ In 2014, an estimated 430,000 MR procedures took place in health facilities in Bangladesh.^3^ Family planning services are offered following MR procedures, but service quality and the range of available methods vary.^11^ Furthermore, some women report not wanting to make a decision about contraception on the day of their procedure.^12^

Mobile phones have the potential for rapidly delivering targeted communications to large and disparate populations at low cost. Evidence shows that mobile phones for health (mHealth) can be effective at changing behavior and improving health outcomes,^13^ and they are increasingly being used by sexual and reproductive health programs to support clients. In recent years, mHealth interventions have been used to promote safer sex and partner notification of sexually transmitted infections,^14^^,^^15^ increase contraceptive use,^16^ support antiretroviral adherence and clinic attendance among people living with HIV,^17^^,^^18^ reduce HIV transmission risk among male sex workers,^19^ support women through the home phase of medical abortion,^20^ and provide support for pregnant women and new mothers.^21^^,^^22^ Some of these intervention types are still at the feasibility or pilot stages, but others have been scaled up. One of the scaled-up interventions is the Mobile Alliance for Maternal Action (MAMA) approach, which started in 2010 and uses a range of mobile technologies (including text messages, voice calls, and apps) to support maternal and newborn health. By 2016, it had 1.9 million subscribers in Bangladesh, 600,000 in India, and 500,000 in South Africa.^22^

Moderate-quality evidence indicates that text messages are effective in supporting antiretroviral adherence and clinic attendance for HIV and sexually transmitted infections.^18^^,^^23^ In many other areas of mHealth for sexual and reproductive health, however, the evidence is limited, and mixed and more rigorous trials are needed.^16^^,^^18^^,^^24^ With respect to contraception, text message interventions in the United States have successfully increased pill continuation and attendance for injectable appointments, interactive voice messages with counselor phone calls have increased use of LARCs among abortion clients in Cambodia, and phone-based information sessions have increased effective contraceptive use among postpartum clients in Ecuador; however, other interventions have had no effect on contraceptive outcomes or have improved knowledge but failed to change behavior.^16^^,^^25^^,^^26^ Feasibility research exploring the use of text message contraceptive reminders among MR and postabortion care (PAC) clients in Bangladesh had promising results, although some concerns were raised about unintended recipients seeing text messages.^27^

Some evidence shows that communication about sexual and reproductive health and access to services can precipitate intimate partner violence (IPV) toward women in certain relationships.^28^^,^^29^ Furthermore, some studies suggest that women's empowerment (e.g., through increased earnings) can more broadly result in increased IPV as new roles are negotiated, but the findings on this topic are mixed.^30^ With respect to phone interventions, a systematic review published in 2013 investigated the effect of mHealth on gender relations and highlighted cases of resulting domestic dispute; however, the authors reported that data were very limited, noting their suspicion that this outcome is not routinely being measured.^31^

Communication about sexual and reproductive health and access to services can precipitate IPV toward women in certain relationships.

More broadly, evaluations of public health interventions often fail to assess potential harms and their underlying mechanisms.^32^ Randomized controlled trials (RCTs) evaluating mHealth family planning interventions have not reported any adverse effects; however, few trials report measuring negative outcomes.^8^^,^^25^^,^^33^^–^^41^ Recent systematic reviews of family planning interventions suggest that impacts on partner relations (including violence) are not routinely measured in this field.^16^^,^^42^ Monitoring adverse events, and specifically IPV, remains important when evaluating mHealth interventions for reproductive health.

**FIGURE 1:**
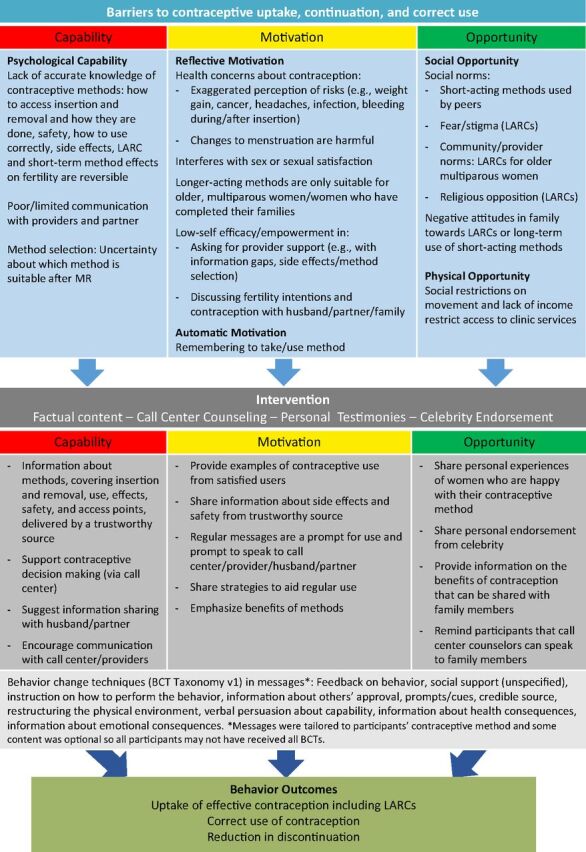
Conceptual Framework for the Mobile Phone Intervention Designed to Support Post-Menstrual Regulation Contraceptive Use in Bangladesh Abbreviations: LARC, long-acting reversible contraceptive; MR, menstrual regulation.

Given the success of the Cambodian intervention and largely positive findings of the feasibility study in Bangladesh, 2 NGOs designed an enhanced mHealth intervention to support post-MR contraceptive use in Bangladesh, with a focus on overcoming barriers to LARC use. The initiative was a partnership between Marie Stopes Bangladesh (MSB), which operates private reproductive health clinics (140 at the time of implementation), and Ipas Bangladesh, which supports government facilities and clinics within the nongovernmental Reproductive Health Services Training and Education Program (RHSTEP) in provision of MR, PAC, and family planning through provider training. The intervention comprised automated interactive voice messages sent to women's mobile phones in the 4 months after MR. The Cambodia intervention relied heavily on call center counseling, and the cost of this component has been cited as a possible barrier to scale-up.^43^ We explored whether an intervention containing more detailed automated content about contraception could be effective. We hypothesized that women who receive a multicomponent mHealth intervention will have a higher rate of LARC use at 2 weeks, 4 months, and 12 months after MR, compared with women who do not receive the mHealth intervention. We also hypothesized that the intervention would increase use of any modern method, reduce subsequent pregnancy or MR, and reduce discontinuation among existing users. The conceptual framework (Figure 1) drew on the COM-B model of behavior change.^44^ The aim of this study was to evaluate the effect of the intervention on contraceptive use and to monitor for adverse events, including IPV, which is widespread in Bangladesh; in 2015, 50% of ever-married women reported having experienced physical violence and 27% sexual violence by their current or previous husband.^45^

This study evaluated the effect of the mHealth intervention on contraceptive use and monitored for adverse events, including IPV.

## METHODS

### Study Design

This study was a single-blinded, multisite RCT of an interactive voice message intervention delivered via mobile phone designed to support post-MR contraceptive use. Women were recruited from 41 health facilities sampled from 93 eligible clinics: clinics located in Chittagong, Dhaka, or Sylhet division (chosen for having lower modern contraceptive prevalence than other divisions at 47%, 54%, and 41%, respectively),^1^ were an MSB facility or a government facility supported by Ipas Bangladesh, had a minimum monthly MR case load of 20, and had no other intervention study underway at the start of recruitment. Facilities were stratified according to size: primary clinics were located in rural and peri-urban areas; secondary clinics, located in urban areas, were larger and provided a wider range of services; and tertiary facilities were hospitals located in large cities. Seventeen units of 19 women were sampled from each strata (N=969 women), using probability proportional to MR client load with some higher-volume facilities being sampled multiple times. During recruitment some clinics had lower client flow than expected and an additional 4 units were sampled to enable the sample size to be reached during the recruitment period. Twenty-five of the sampled clinics were MSB and 16 were government and nongovernment RHSTEP facilities supported by Ipas Bangladesh. Thirteen sampled clinics were in Chittagong, 23 in Dhaka, and 5 in Sylhet. The trial protocol was published in 2017.^46^ Ethical approval was received from the Bangladesh Medical Research Council, the London School of Hygiene and Tropical Medicine Research Ethics Committee, the Marie Stopes International Ethical Review Committee, and the Population Council Institutional Review Board.

### Participants

Women were eligible to participate if they had had an MR procedure from a participating clinic during the recruitment period, were 18 to 49 years of age, did not receive general anesthesia for their MR procedure (since recruitment took place immediately after the MR procedure at which time general anesthetic clients may not have been well enough to give informed consent), were physically and emotionally able to consent, reported that they had a personal mobile phone, consented to receive voice messages about family planning on their phone, and did not intend to become pregnant or use a permanent method of contraception in the next 6 months. The latter group were excluded as the intervention focused on supporting uptake and continuation of and switching between contraceptive methods, which was information not deemed to be relevant to women using permanent methods. The study was introduced to women by the clinic provider after completing MR services. Interested participants were referred to a clinic-based research assistant (RA) for screening and recruitment, which took place within 2 days of the MR. All participants gave written informed consent in the presence of a witness of their choice.

**FIGURE 2:**
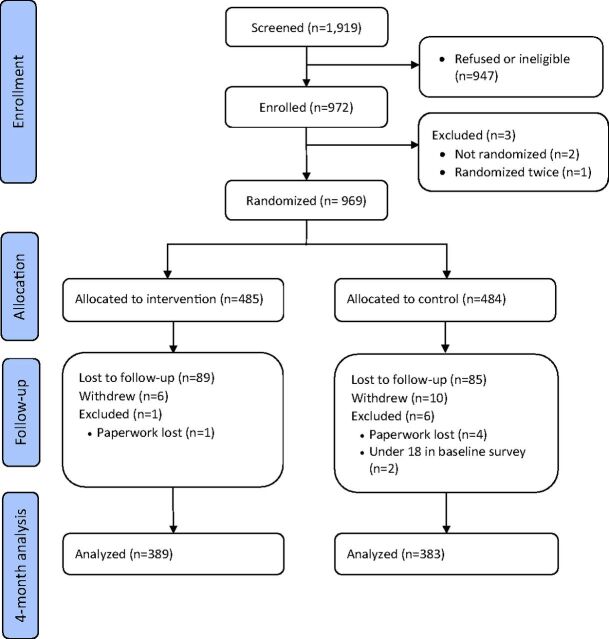
Flowchart of Participants in a Randomized Controlled Trial of a Mobile Phone Intervention Designed to Support Post–Menstrual Regulation Contraceptive Use in Bangladesh (2015–2016)

Study recruitment ran between December 19, 2015, and March 1, 2016. We screened 1919 MR clients, 947 refused to take part or were ineligible, and 972 were recruited (Figure 2). We do not have a breakdown of the number according to refusal or ineligibility. We also do not have quantitative data on the reasons for refusal or ineligibility; however, RAs reported that the most common reasons for nonparticipation were refusal due to not having time, concern from the woman that her family would find out about her MR, and lack of approval from the woman's husband. The most common reasons for ineligibility were wanting to conceive within the next 6 months, planning to use sterilization, and having received general anesthesia for the MR procedure.

### Randomization and Masking

Participants were allocated with a 1:1 ratio to the intervention (mobile phone interactive voice messages) or control group (no voice messages). The allocation sequence was generated remotely prior to the start of enrollment and kept by an independent researcher based in Dhaka using the Microsoft Excel formula “=RANDBETWEEN(1, 2)”. No minimization was used. Allocation concealment was achieved via the following steps:

1. Participants were entered into the mHealth platform by recruiting RAs using a secure app following enrollment.
2. At the end of each day, a list of new participant IDs was downloaded from the system by a technical officer at MSB and sent to the independent researcher, who had no contact with RAs or participants.
3. The independent researcher assigned the IDs sequentially to the pregenerated allocation list.
4. The independent researcher sent the ID list with allocations to the technical officer, who activated calls for intervention participants.

RAs recruiting and interviewing participants, clinic staff, and participants were not informed of participant allocations. Participants were told that if they were in the intervention group they would start receiving messages within a few days of recruitment.

A total of 969 participants were randomized correctly, 485 and 484 to the intervention and control groups, respectively. Enrollment paperwork was lost for 5 participants, and 2 participants were excluded at baseline due to age ineligibility.

### Procedures

All participants received existing standard care at the clinic, which included family planning counseling and offer of available methods, and were provided with the number of a paramedic-led reproductive health call center operating 24/7 and established at MSB for the study. Calls to the call center were charged at the price of a call to a mobile phone. Intervention participants were sent automated interactive voice messages to their mobile phone over the 4-month period after their MR; the intervention is summarized in Box 1 and full details are given in Supplement 1. Sample message content is provided in Box 2 and all messages are included in Supplement 2. Control participants were not sent any voice messages. Participants were interviewed in person at baseline and via phone at 2 weeks and 4 months post-MR. Responses were recorded on paper at the time of interview and entered into a Microsoft Access database by a data entry clerk at a later date. RAs conducting follow-up interviews were blinded to allocation at the start of each interview. Participants were reimbursed the following amounts for starting an interview: 300 Bangladeshi taka (BDT) (US$3.84 in December 2015) at recruitment, 100 BDT (US$1.28) at the 2-week follow-up, and 200 BDT (US$2.56) at the 4-month follow up. Participants could withdraw from the study during interviews or at any time by calling the study number or call center.

BOX 1 **Summary of the Mobile Phone Intervention**

**Intervention Development and Aims**

The intervention development drew on formative and feasibility research conducted in Bangladesh,^16^^,^^22^ behavior change theory,^20^ and literature on evidence for mHealth globally and barriers to contraceptive use in this population.^14^ The aim of the intervention was to address information gaps and misconceptions about contraception, particularly for long-acting reversible contraceptives (LARCs), increase motivation for family planning uptake among nonusers, support continuation among users, and encourage method switching for those unhappy with their method.

**Intervention Structure**

The intervention comprised 11 interactive voice messages delivered to the participant's mobile phone over the 4 months post-menstrual regulation (MR). The first 7 messages were delivered at weekly intervals and the remainder at fortnightly intervals. Participants were asked at recruitment to select 1 of 5 time slots when they would prefer to receive the messages. When the message was sent, if switched on and in network coverage, the phone would ring. If it was answered, the automated content would start to play. If the message was not delivered or answered it would be resent twice after 30-minute intervals. After 3 failed attempts, no further calls would occur until the next scheduled message. It is uncommon in Bangladesh for mobile phones to have voicemail capacity, so messages were not stored for later access.

**Intervention Content**

Seven generic messages were sent to all clients, aiming to increase motivation for contraceptive use and address common fears and information gaps, particularly for LARCs, and 4 messages were tailored to the method of contraception (or no method) chosen by the participant after their MR procedure. If use of a different method was reported in the 2-week survey or to the call center, the woman's message group was updated and messages restarted. Consequently, women who switched methods received more than 11 messages over a duration longer than 4 months. Messages included factual information delivered by a “doctor apa” (female doctor), 2 personal stories in which women told of their experience using an implant or IUD, and a message recorded by a local celebrity endorsing LARCs.

**Interactive Features**

Each message ended with a menu allowing participants to use their keypad to repeat the initial content, listen to further content (a factual message about each modern method of contraception available in Bangladesh), connect to the call center to receive personalized counseling, indicate they did not need further information at that time, or opt out from receiving further messages.

**Cost and Management of the Intervention**

Messages, including connecting calls to the call center, were free to call recipients. The intervention was built and hosted by “I am digital” (Dhaka), and messages were scheduled to be sent out on a daily basis by a technical officer based at MSB in Dhaka. Members of the study team were enrolled into the intervention to monitor its functioning.

For additional details about the intervention, see Supplement 1.

BOX 2 **Sample Interactive Voice Message Content**

**Pill Message 1 (sent to pill users only)**

Hello, this is your doctor apa calling to congratulate you for choosing pills! Pills are effective in preventing pregnancy if taken at the same time every day. Remember that to work, pills must be taken every day whether or not you have sex. If you miss a pill, always take one missed pill immediately, then continue with the pills in the pack on your normal schedule. If you miss three or more pills in a row, you are at risk for pregnancy so use condoms for seven days and consider using emergency contraception. If you have questions or concerns about using pills correctly, we are available 24 hours a day to give advice. *To listen to the message again, press 1. To learn more about family planning methods, press 2. Press 3 to talk to a counselor. If you don't need any information now, press 4. Press 5 to stop receiving these messages.*

**Implant Personal Experience Message (sent to all participants)**

Hello, my name is Mithila, and I'm calling as part of your doctor apa program to tell you why I chose to use an implant. We have been married for three years, and we knew we wanted to have children, but we didn't want them straight away. I started using the pill, but I was not very good at remembering to take it every day, which made me worry about getting pregnant. I decided to visit a provider at a nearby health center, who told me that the implant would protect me for up to three years and I would be free from the worry of unintended pregnancy. I was nervous about having something placed under the skin of my arm, but the doctor showed me where it would be placed, where it will remain, and how it will protect me from pregnancy. The insertion was quick—it only took about five minutes, and then I didn't have to worry about it anymore. My bleeding got a bit lighter after the first few months. Last year, I decided to have my implant removed, and within three months I was pregnant. Now that our baby is born, I am preparing to get an implant again. If you're interested in more facts about the implant, speak to a service provider or visit the hospital. *To listen to the message again, press 1. To learn more about family planning methods, press 2. Press 3 to talk to a counselor. If you don't need any information now, press 4. Press 5 to stop receiving these messages.*

For all message content, refer to Supplement 2.

### Procedures to Minimize the Risk of Harm

The study was designed with the following procedures to minimize the risk of harm. Many women in Bangladesh report needing to obtain permission from their husband before making decisions.^47^ At recruitment, women were given the additional options of enrolling with their husband or other individual of their choice, discussing the study with others before enrolling, having the RA call their husband or someone else to explain the study, and enrolling at a location of their choice. During enrollment, women listened to an example of an automated voice message about family planning and were asked if receiving similar messages on their phone was acceptable to them, what would happen if their husband or partner heard the message, and what would happen if someone else such as an in-law or other family member heard the message. If concerns were raised, the participant was advised not to participate. The intervention did not mention MR or abortion, the participant's visit to a clinic, or the source of the messages being MSB or Ipas Bangladesh. At the end of each automated voice message, participants had the option of pressing 5 on their key pad to opt out from getting further messages. They could also opt out of the intervention by calling the call center or study number, both of which they received at recruitment.

### Outcomes

The primary outcome was self-reported LARC (IUD or implant) use at 4 months post-MR. Secondary outcome measures were use of a LARC at 2 weeks; use of any effective modern contraceptive method, defined as methods with <10% 12-month pregnancy rate (pill, injectable, implant, IUD, or sterilization), at 2 weeks and 4 months post-MR; and subsequent MR, pregnancy, adverse events including experience of violence, or contraceptive discontinuation at any point during the 4-month intervention period. Contraceptive discontinuation was measured using an adaptation of the Demographic and Health Survey contraceptive calendar.^1^ At 4 months, participants were also asked about intervention use. Adverse events were measured using an open question: “Did anything happen to you as a result of you being in this study? Good or bad?” Participants who said yes were asked what happened. In addition, violence was measured using 3 closed questions that named specific acts:

1. Physical IPV: “In the last 4 months since the MR when you joined this study, has your husband/partner hit, kicked, slapped, or otherwise physically hurt you?”
2. Sexual IPV: “In the last 4 months since the MR when you joined this study, has your husband/partner physically forced you to have sexual intercourse with him even when you did not want to?”
3. Physical violence perpetrated by the participant's in-laws: “In the last 4 months since the MR when you joined this study, have your in-laws hit, kicked, slapped, or otherwise physically hurt you?”

No data and safety monitoring board was set up for this study because the intervention was behavioral, not clinical, and the negative effects of mHealth were thought to be minimal. The ethics committees that reviewed the study and the STEP-UP Consortium Advisory Group advised on safety aspects when follow-up data were analyzed.

### Statistical Analysis

Using data from MSB and government clinics supported by Ipas Bangladesh, we estimated that 9.4% of control participants would be using a LARC after 4 months. Therefore, assuming equal numbers of women in the intervention and control group, we calculated that a minimum of 960 participants would be required to detect an increase in LARC use of 7% in the intervention group to 16.4% at 4 months of follow-up (a relative risk of 1.74), with .05 significance level and 80% power, allowing for 25% loss to follow-up. A similar intervention in Cambodia revealed an increase in LARC use from 9% to 29% after 4 months;^8^ however, a smaller difference could be important given the low cost of sending phone messages.

We conducted analyses on an intention-to-treat basis using Stata version 14.2 (StataCorp LLC, College Station, TX) and followed the prespecified analysis plan.^46^ Two independent analyses were conducted, and one analyst [JC] was blinded to the allocation groups. We calculated the effect of the intervention on the primary and secondary outcomes as an odds ratio with a 95% confidence interval using multivariate logistic regression to allow us to adjust for any baseline imbalances among prespecified variables that we hypothesized may be associated with the outcomes: baseline method use (method taken on the day of the MR) or experience of violence in the past year, age, and socioeconomic status (SES) measured using the Poverty Probability Index (PPI).^48^^,^^49^ We used multiple imputation (MI)^50^ to impute missing outcome and baseline data separately for the 2 allocation groups during analysis of the primary outcome: we used baseline, 2-week, and 4-month LARC use; baseline age; and SES (PPI score) to impute 100 data sets.^48^ We then fitted the logistic regression of 4-month LARC use on allocation group and baseline LARC use to each imputed data set and combined the results for final inference using Rubin's rules.

As a check on our imputation analysis, we conducted logistic regression of 4-month LARC use on allocation group without multiple imputation, adjusting for age, SES (PPI score), and baseline LARC use. This analysis therefore makes a slightly stronger missing at random assumption than the MI analysis. Where there was some evidence (from a simple association test) for an intervention effect on a secondary outcome, we conducted a model-based analysis using MI (which was not specified in the analysis plan). The MI model included the secondary outcome under consideration; intervention; baseline use/experience of secondary outcome being investigated; baseline, 2-week, and 4-month LARC use; baseline age; and SES (PPI score). Logistic regression of secondary outcome on intervention and baseline was then fitted to each imputed data set, and the results were combined for final inference using Rubin's rules. Because there were imbalances in some baseline data, we carried out additional logistic regression of imputed data sets, adjusting for baseline variables that were prespecified in the protocol or where there was a significant difference between groups: use or experience, age, SES, and education.

We carried out a subgroup analysis to examine any difference in effect of allocation on the primary outcome using logistic regression controlling for age, SES, and baseline use where these were not subgroup categories. Six subgroups were prespecified, and 1 was added during analysis (facility type). We assessed contraceptive discontinuation among participants who started using an effective contraceptive method during the first 4 weeks post-MR, using Kaplan-Meier survival analysis and the log rank test. We defined discontinuation as stopping a method and not starting another effective modern contraceptive method within 2 weeks. We present curves by method for the pill and injectable individually and a combined curve for LARCs due to the low numbers of participants who discontinued these methods. We carried out an additional (not prespecified) subgroup analysis to examine any difference in effect of allocation on physical IPV using logistic regression controlling for age, SES, and baseline experience of physical IPV where they were not subgroup categories.

The trial has been registered with ClinicalTrials.gov (NCT02579785) since October 16, 2015.

### Role of the Funding Source

The funder of the study had no role in study design, data collection, data analysis, or data interpretation or in writing of the report. The corresponding author had full access to all study data and had final responsibility for the decision to submit for publication.

## RESULTS

We analyzed baseline data for 962 participants (Table 1). Follow-up was completed in July 2016 and primary outcome data were collected and used from 772 participants, an 80% follow-up rate; 389 (80% follow-up rate) from the intervention arm, and 383 (79% follow-up rate) from the control arm.

**TABLE 1.** Baseline Characteristics of the Intention-to-Treat Population

	Intervention Arm (n=484)	Control Arm (n=478)
**Age, years, mean (SD)**	28 (6)	28 (6)
Missing,^a^ No. (%)	1 (<1)	0 (0)
**Education, No. (%)**		
Up to end of primary	150 (31)	115 (24)
Over primary	334 (69)	362 (76)
Missing^a^	0 (0)	1 (<1)
**Poverty status (PPI), No. (%)**		
Likelihood of living below US$1.25	17 (17)	15 (15)
Missing^a^	16 (3)	21 (4)
**Division recruited from, No. (%)**		
Dhaka	286 (59)	295 (62)
Chittagong	141 (29)	127 (27)
Sylhet	57 (12)	56 (12)
**Location of residence, No. (%)**		
City	173 (36)	182 (38)
Town	119 (25)	120 (25)
Village	192 (40)	175 (37)
Missing^a^	0 (0)	1 (<1)
**Number of living children, No. (%)**		
0	67 (14)	58 (12)
1–2	280 (58)	292 (61)
≥3	137 (28)	127 (27)
Missing^a^	0 (0)	1 (<1)
**Marital status, No. (%)**		
Married or cohabiting	478 (99)	470 (98)
Separated/divorced/widowed	1 (<1)	3 (<1)
Never married or cohabited	5 (1)	5 (1)
**Religion, No. (%)**		
Islam	437 (90)	438 (92)
Hinduism	38 (8)	30 (6)
Buddhism	8 (2)	7 (1)
Christianity	1 (<1)	3 (1)
**Who makes decision whether participant uses contraception, No. (%)**		
Self only	84 (17)	79 (17)
Self and someone else	337 (70)	346 (72)
Someone else	63 (13)	53 (11)
**Type of MR received at baseline, No. (%)**		
Surgical	353 (73)	348 (73)
Medical	131 (27)	130 (27)
**Chose an effective method of contraception on day of MR, No. (%)**		
Yes, chose a LARC (IUD or implant)	46 (10)	65 (14)
Yes, chose a short-acting method (pill or injectable)	233 (48)	227 (47)
No	203 (42)	186 (39)
Missing^a^	2 (<1)	0 (0)
**Facility type, No. (%)**		
Public-sector clinic supported by Ipas Bangladesh	229 (47)	245 (51)
MSB clinic	255 (53)	233 (49%)
**Facility size, No. (%)**		
Primary	172 (36)	148 (31)
Secondary	155 (32)	171 (36)
Tertiary	157 (32)	159 (33)
**Experience of physical IPV in the last year, No. (%)**		
Yes	58 (12)	65 (14)
No	425 (88)	411 (86)
Missing^a^	1 (<1)	2 (<1)
**Experience of sexual IPV in the last year, No. (%)**		
Yes	123 (25)	129 (27)
No	361 (75)	346 (73)
Missing^a^	0 (0)	3 (<1)
**Experience of violence perpetrated by in-laws in the last year, No. (%)**		
Yes	13 (3)	20 (5)
No	440 (97)	416 (95)
Missing^a^	31 (6)	42 (9)

Abbreviations: IPV, intimate partner violence; IUD, intrauterine device; LARC, long-acting reversible contraception; MR, menstrual regulation; MSB, Marie Stopes Bangladesh; PPI, Poverty Probability Index; SD, standard deviation.

a Not included in denominator for calculation of percentages.

No evidence was found for an effect of the intervention on the primary outcome of LARC use at 4 months post-MR: 48 (12%) of intervention participants and 59 (15%) of control participants reported using a LARC at 4 months (*P*=.22). After imputing missing data, we found no evidence that participants in the intervention arm had higher odds of using a LARC than women in the control arm (adjusted odds ratio [aOR]=0.95; 95% confidence interval [CI]=0.49 to 1.83). The prespecified analysis under a weaker missing at random assumption also revealed no evidence that odds were higher in the intervention arm (aOR=1.06; 95% CI=0.53 to 2.13). There was also no evidence for an effect of the intervention on use of any effective modern contraception at 4 months, subsequent pregnancy, subsequent MR, or on LARC and any effective modern method use at 2 weeks post-MR (Table 2).

**TABLE 2.** Effect of the Mobile Phone Intervention on Primary and Secondary Outcomes at 2 Weeks and 4 Months Follow-Up

Outcome	Intervention Arm n/N (%)	Control Arm n/N (%)	*P V*alue (χ^2^)	Unadjusted OR (95% CI)	Adjusted OR (95% CI)^a^
**Primary outcomes**					
LARC use at 4 months	48/389 (12%)	59/383 (15%)	.22	0.77 (0.51, 1.17)	1.06 (0.53, 2.13)^b^
LARC use with MI at 4 months (100 imputations)	55/484 (11%)	72/478 (15%)	.11	0.73 (0.49, 1.08)	0.95 (0.49, 1.83)^c^
**Secondary outcomes**					
***4-month follow-up***					
Effective modern method use (any method)^d^	214/389 (55%)	204/383 (53%)	.63	1.07 (0.81, 1.42)	1.04 (0.75, 1.43)^b^
Subsequent pregnancy	6/389 (2%)	10/383 (3%)	.30	0.58 (0.21, 1.62)	0.48 (0.16, 1.43)^e^
Subsequent MR or abortion	2/389 (<1%)	4/383 (1%)	.40	0.49 (0.09, 2.69)	0.43 (0.08, 2.41)^e^
***2-week follow-up***					
LARC use	48/413 (12%)	56/411 (14%)	.39	0.83 (0.55, 1.26)	1.57 (0.68, 3.62)^b^
Effective modern method use	223/413 (54%)	221/411 (54%)	.95	1.01 (0.77, 1.33)	1.07 (0.74, 1.54)^b^
***4-month contraceptive calendar (log rank test)***	Discontinuations/time at risk (weeks)	Discontinuations/time at risk (weeks)	Log rank test		
LARC discontinuation	2/661 (<1%)	2/844 (<1%)	.81		
Injectable discontinuation	20/620 (3%)	14/517 (<3%)	.56		
Pill discontinuation	27/1693 (2%)	32/1555 (2%)	.33		
**Adverse outcomes at 4 months**					
Physical IPV	42/386 (11%)	25/382 (7%)	.03	1.74 (1.04, 2.92)	1.97 (1.12, 3.46)^b^
Physical IPV with MI (100 imputations)^f^	57/484 (12%)	31/478 (6%)	.03	1.87 (1.07, 3.27)	2.16 (1.16, 4.02)^c^
Sexual IPV	47/386 (12%)	36/379 (10%)	.23	1.32 (0.83, 2.09)	1.25 (0.78, 2.02)^b^
Physical violence from in-laws	6/384 (2%)	4/380 (1%)	.54	1.49 (0.42, 5.33)	1.20 (0.31, 4.62)^b^
**Anything happened to them as a result of being in the study**					
Nothing	335/385 (92%)	366/381 (96%)	.06		
Something good	28/385 (7%)	13/381 (3%)		2.22 (1.13, 4.36)	2.25 (1.14, 4.44)^g^
Something bad	2/385 (<1%)	2/381 (<1%)		1.03 (0.14, 7.36)	2.10 (0.19, 3.60)^g^

Abbreviations: CI, confidence interval; IPV, intimate partner violence; IUD, intrauterine device; LARC, long-acting reversible contraception; MI, multiple imputation; MR, menstrual regulation; OR, odds ratio; PPI, Poverty Probability Index; SES, socioeconomic status.

a Total number varies due to missing data on baseline covariates included in the model (see Table 1).

b Adjusted for baseline use of these methods/experience of this outcome, age, and SES (PPI Score).

c Adjusted for baseline use/experience only.

d Pill, injection, implant, IUD, or sterilization. At 4 months, participants were asked about how regularly they take the pill and were only classified as pill users if they reported always or usually taking it on time.

e Adjusted for baseline LARC use, age, and SES (PPI score).

f Analysis not specified in study protocol.

g Adjusted for age and SES (PPI score).

No evidence was found for an effect of the intervention on the primary outcome of LARC use at 4 months post-MR.

The log rank test indicated that there was no evidence for a difference in contraceptive discontinuation rates between the allocation groups (Supplement 3). We found no evidence for a difference in effect of the intervention on LARC use between subgroup categories (Supplement 4).

The intervention appeared to have an effect on reported physical IPV measured using a closed question asking about specific acts (Table 2): 42 (11%) of participants in the intervention arm reported physical IPV in the 4 months post-MR versus 25 (7%) in the control arm (aOR=1.97; 95% CI=1.12 to 3.46; *P*=.03). A post-hoc analysis using MI (under a weaker missing at random assumption) to handle missing data found participants in the intervention group had 2.16 (95% CI=1.16 to 4.02) times higher odds of reporting physical IPV as participants in the control group. There was no evidence in crude or adjusted models that the intervention had an effect on self-reported sexual IPV (47 [12%] vs. 36 [10%]; aOR=1.25; 95% CI=0.78 to 2.02; *P*=.23) or self-reported physical violence perpetrated by in-laws (6 [2%] vs. 4 [1%]; aOR=1.20; 95% CI=0.31 to 4.62; *P*=.54). Reports of adverse outcomes also did not differ when participants were asked whether something happened to them as a result of participating in the study: only 4 participants (2 per group) reported something negative, and all 4 reported pain or other effects of the MR or contraception. Additional analysis of imputed data sets adjusting for imbalances in baseline data had no effect on the scientific conclusions. We found no evidence for a difference in effect of the intervention on physical IPV between subgroup categories (Supplement 5).

The mHealth intervention had an effect on reported physical IPV.

During the 4-month follow-up survey, 319 (85%) intervention and 89 (25%) control participants reported that they received voice messages about family planning since they joined the study. Among intervention participants who received messages, 170 (54%) said they listened to all the messages, 141 (45%) listened to some, and 5 (2%) listened to none. A small number of participants (12 intervention and 1 control participant) reported that they pressed 5 to opt out from receiving further messages.

## DISCUSSION

Women allocated to receive interactive messages about contraception (the intervention group) were more likely to report physical IPV compared with the control group receiving usual care, but there was no evidence of an effect on self-reported LARC or effective modern contraceptive use. The increase in reported physical IPV was observed when measured using a direct question that named specific acts of violence, but no conflict was reported in response to an open question about positive or negative effects of being in the study.

Women who received the messages about contraception reported more physical IPV; LARC and effective modern contraceptive use were not affected.

### Strengths and Limitations

A key strength of this study is the randomized controlled design, which allowed us to identify an increase in a rare negative outcome; it is unlikely this effect would have been detected in a noncomparative study. The randomization process was conducted remotely, and clinic and research staff involved in recruitment were blinded to allocation as was 1 analyst. While some bias may have been introduced through lack of full blinding in the 4-month survey, steps were taken to ensure that RAs conducting this follow-up interview were blinded during reporting of primary and secondary outcomes, which were placed at the beginning of the questionnaire. Loss to follow-up was relatively small and was similar across arms, and MI (assuming missing at random) was used to handle missing data.

Table 1 shows that baseline differences existed between the 2 allocation groups, which occurred by chance during the randomization process. We adjusted for these differences, and although some residual confounding may have remained, it is very unlikely to affect the results.^51^ Notably, a higher proportion of patients in the control arm than the intervention arm were using LARCs at baseline (14% vs. 10%, respectively). This is the principal reason why the point estimate from adjusted analysis for LARC use at 4 months and 2 weeks is greater than the corresponding unadjusted point estimate. Given the baseline imbalance, the adjusted results are preferred; however, the difference between the 2 must be interpreted cautiously as the 95% CI from the adjusted analysis includes the unadjusted estimate in each case.

The proportion of control participants using a LARC at 4 months (15%) was higher than predicted during sample size calculations, slightly reducing the power; however, the loss to follow-up was less than expected. Post-hoc power calculations indicate that we had a sample size that was large enough to detect an increase of 9 percentage points at 4 months of follow-up, from 15% in the control group to 24%, and of 8 percentage points to 23% when using multiple imputation. The study was not powered for the subgroup analyses, so we were unlikely to find any significant results in these analyses; however, the results do not appear to explain the increase in IPV.

The main limitation of this study is that all outcomes are self-reported and may have been affected by a number of types of reporting bias; however, self-reporting is standard for trials of interventions to support contraceptive use.^8^ Recall bias is likely to have affected both allocation groups in the same way, but social desirability bias may have had a differential effect; for example, participants in the intervention group may have felt more pressure to report something positive happened to them as a result of study participation because they received the intervention. Another possibility is that the intervention may have increased trust in the study team, leading intervention participants to feel more able than control participants to report violence.

Control participants were not sent placebo or dummy messages to their phone without active content, which limits our ability to identify whether the increase in IPV was a response to the message content or to getting messages at all. However, given that both the message content and format were integral to the intervention design, we do not consider this as limiting our finding that this mHealth intervention led to an increase in violence. A noteworthy proportion of control participants reported receiving messages about family planning during the study period; it is not clear if these were messages from another source, or whether some participants considered the reminders about the follow-up interviews as family planning messages. Initial analysis of interactive voice response system process data suggest minimal contamination was present, but this possibility is being explored further.

The measures of violence used did not capture frequency or severity, and addition of these outcomes may have provided more insight into the relationship between the intervention and this adverse outcome.

The measures of violence used did not capture frequency or severity.

### Discussion in Relation to Existing Literature

No adverse outcomes were reported in a similar study in Cambodia, which improved contraceptive use among postabortion clients using interactive voice messages^8^; however, only a single open question was used: “Did anything happen to you ‘positive or negative’ as a result of participating in the trial?” (personal communication with the study principal investigator). In an RCT evaluating instant messages delivered by a mobile phone app designed to increase the acceptability of contraception among young people in Tajikistan, 4 of 470 participants interviewed at follow-up reported experiencing physical violence since being in the study, but there was no evidence for a difference between the control and intervention groups.^34^ A trial of text message support for adolescents and young adults in the United States using an injectable contraceptive stated that no adverse events were reported by participants; however, no information was given on how such events were measured.^35^ Other trials of mHealth interventions for contraception have not reported on conflict or violence.^25^^,^^33^^,^^36^^–^^41^ A 2013 systematic literature review of the effects of mHealth interventions and interventions aimed at increasing mobile phone ownership and use among women on gender relations in developing countries found evidence for both positive and negative effects.^31^ The included literature suggest that mobile phone interventions can increase women's decision-making power and social status and can increase male participation in health areas usually targeted to women.^31^ However, in a study in which participants had been given phones as part of the intervention, some tensions over phone use were reported, and an intervention supporting mobile retail business among women led to tension and abuse when a participant's husband's role as the highest earner was challenged.^31^ The authors identified only 7 articles on this topic that met their inclusion criteria, and they concluded that effects of mHealth on gender relations are often not being measured.^31^ Qualitative research in Ghana conducted between 1994 and 1996 found that in addition to having many positive effects, the introduction of family planning services in the region led to tensions in gender relations.^29^ For example, some men reported fears that women using contraception will be unfaithful. There are anecdotal reports of similar attitudes in Bangladesh.

### Meaning and Mechanisms

Although a possibility, it is unlikely that the increase in self-reported IPV is a chance finding: the *P* value of .03 equates to a 1 in 33 chance. As discussed in the limitations section, the result could possibly be due to reporting bias if receipt of the intervention made women feel more able to disclose IPV. The result may reflect a true increase in IPV by one of the following mechanisms. It is plausible that although participants agreed to messages being sent to their phone about contraception and were played an example at recruitment, messages were more troublesome when received at home: messages may have been overheard by others who found the content to be unacceptable, messages may have disclosed contraceptive use, or they may have led to suspicion of infidelity. The messages did not mention the participant's MR or study participation; however, such information may have been revealed if she was questioned about the calls. Notably, concern about family finding out about an MR was a common reason for potential participants refusing to take part in the study.

It is unlikely that the increase in self-reported IPV is a chance finding.

In Bangladesh, not seeking a husband's permission before making a decision is often reported to warrant wife-beating,^47^ and a husband's lack of approval was also given as a reason for refusal to participate in the study. Women were given the opportunity of enrolling with their husband or of informing him before they agreed to participate, but some of those who did not get their husband's consent could have experienced IPV. Conflict may also have resulted from increased phone use, from calls coming from an unknown number, or from the timing or repetitive nature of calls. IPV may plausibly occur in interventions that empower women to use contraception, thereby challenging existing power dynamics; however, there is no evidence to support this mechanism in this trial.

No participant reported violence in response to the open question about study effects, and intervention participants were more likely than control participants to report a positive experience. This result may reflect acquiescence bias, social desirability bias, or concerns that the conversation was being overheard. It is also possible that participants did not attribute violence that they may have experienced to the intervention. Reported levels of violence were higher at baseline than at 4 months but this is to be expected for the following 2 reasons. First, different time frames were used for the baseline and 4-month surveys; at baseline participants were asked about violence in the past year in order to be able to compare the level of violence in this population with other surveys and because a 12-month follow up was planned, as explained below. At follow-up, participants were asked about violence during the intervention period—the past 4 months—in order to examine any difference in relation to study participation. Second, there is evidence demonstrating unintended pregnancy and abortion are associated with partner violence. Therefore, we may expect to see higher than normal levels of violence when interviewing women at the time of an MR procedure.^51^

The study protocol included a quantitative 12-month phone follow-up and in-depth interviews with a small number of participants.^46^ The 4-month primary outcome data were analyzed before the 12-month survey was conducted in order to inform programming at MSB. After observing the increase in physical IPV and the lack of benefits from receiving the intervention, the findings were shared with the 4 ethics committees who had approved the protocol. A decision was made not to proceed with the 12-month survey due to safety concerns; although the intervention had ended, it was unclear whether the study participation itself was a potential cause of risk. The in-depth interviews, which had been planned as part of a mixed-methods process evaluation, were redesigned to explore the violence outcome and to increase safety protocols when recontacting and interviewing trial participants. The interviews, conducted in 2017, are currently being analyzed and full results will be reported elsewhere. No in-depth interview participant reported conflict or violence resulting from study participation. However, the possibility exists that anyone who experienced conflict may not have agreed to an in-person interview. In the interviews, women reported that phone sharing with husbands was common, so the possibility of messages being overheard was high. There was some evidence of women's phone calls being monitored, and there were a few cases of marital conflict due to phone use more broadly. A number of participants reported not talking about the study due to concern that it would result in other family members or people in the local community finding out about their MR. The majority of participants interviewed had informed their husband about their MR and the study, but many had not shared this information with their in-laws or others. Many reported opposition to MR among other family members and the wider community. The lack of effect of our intervention on contraceptive use is also important. This finding contrasts with results from voice call interventions that increased contraceptive use among postabortion clients in the study in Cambodia discussed earlier and among postpartum clients in Ecuador.^8^^,^^26^ The lack of effect in our study may be due to differences in the intervention design, the context, or both. Self-reported process data indicate that the majority of participants listened to some or all of the messages, suggesting that the lack of effect was not due to non-receipt of messages. Both the Cambodian and Ecuadorian interventions relied heavily on individualized counseling, and although our messages linked participants to a call center, we aimed to explore the effect of automated content and placed this at the start of the call because counselor-intensive interventions are more costly to provide at scale.^26^^,^^43^ Furthermore in Cambodia, but not in Bangladesh, counselors were able to book clinic appointments, which may have helped to translate behavioral intentions into actions.^43^

Text and voice-based interventions with proven effectiveness have used messages that are short in length,^8^^,^^16^ and recipients have reported that getting only small amounts of information at a time facilitates the assimilation of new content.^15^ In contrast, each of our messages contained many ideas. The temporary nature of automated voice messages may make them unsuitable for delivery of complex information because they cannot be revisited after the call has ended and content may be missed if the call comes at an inconvenient time.

Our messages may not have contained the correct content or a sufficient amount of information to adequately address established individual or social barriers to contraceptive use in this population, such as health concerns.^1^^,^^7^ However, interpersonal factors and social norms may limit the effectiveness of interventions targeting the individual in Bangladesh, where 35% of married women report that decisions about their own health are made mainly by their husband or someone else.^1^ This influence may be particularly relevant for LARCs; in a recent survey in one area of the country, 64% of women reported that their husband disapproves of the implant and the IUD, compared with 8% for the pill.^7^

Twenty-five percent of control participants reported receiving voice messages about family planning during the study period. Prior to the trial, some MSB trial clinics made phone calls to MR participants after their procedure to provide follow-up support, including for contraceptive use. Clinics were asked to stop this practice during the trial, but it is possible that some calls may have been made. Such calls may have weakened any intervention effect.

### Implications for Research and Services

Caution is needed when conducting follow-up of clients who have accessed sexual and reproductive health services, both for programming and research purposes, and with development of other mHealth interventions in this field. A simple call from a provider, rather than automated content, would allow the caller to check who is speaking before revealing their identity and the purpose of the call. The use of “withheld” numbers are recommended for outbound calling as are scripts ensuring confidentiality. Where appropriate, callers may be trained to give a “cover story” in case the call is interrupted. It is vital that mHealth reproductive services are “opt in” and that participants have adequate opportunity to opt out once the intervention has started. Programmers may also consider the possibility of offering alternative delivery mechanisms to women who think their partner may object to the phone calls. With respect to family planning, content that does not reveal contraceptive use may be less personal but could be safer. However, it may be preferable, and still effective,^8^ to avoid any reference to the topic of the call and to use neutral outbound messages only as a way to maximize call center use, particularly when targeting women at a sensitive time in their lives such as post-MR. Studies need to explore how call center counselor-intensive interventions could be financed at scale, for example, via government support, discounted mobile operator charges, corporate sponsorship, and user payment, although the latter must be investigated carefully to ensure it does not negatively affect use or exclude certain groups.^22^ Where smartphones are widely available and illiteracy is not a barrier, apps that can be password protected may offer more options for privacy because women will be able to seek information on their own terms. When developing theoretical frameworks of intended intervention mechanisms, it is also important to consider what unintended and harmful outcomes are possible and how they could occur.^32^ In addition to supporting efforts to reduce intervention risks during the design phase, this consideration can inform the rigorous measurement of potential adverse outcomes and exploration of the mechanisms behind them.^32^

Caution is needed when conducting follow-up of clients who have accessed sexual and reproductive health services.

## CONCLUSIONS

The increase in self-reported violence against women who received automated, interactive voice messages about contraception, despite strong privacy and screening measures, demonstrates the importance of carefully considering the potential for negative outcomes and their underlying mechanisms when developing interventions on sensitive reproductive health topics. The findings also highlight the need to routinely include specific measures with named acts of violence when evaluating mHealth interventions on sexual and reproductive health. We recommend that future mHealth studies set up data safety monitoring boards or make a data and safety monitoring plan to review preliminary effects of the intervention on safety outcomes. Analysis of process data from this trial to explore the mechanism behind the violence outcome and reasons for the noneffect on contraceptive use will be reported elsewhere. That report will include process data from the interactive voice response phone system and from in-depth interviews with study participants.

The study findings highlight the need to routinely include specific measures with named acts of violence when evaluating mHealth interventions on sexual and reproductive health.

## Supplementary Material

19-00015-Reiss-Supplement1.pdf

19-00015-Reiss-Supplement4.pdf

19-00015-Reiss-Supplement5.pdf

19-00015-Reiss-Supplement2.pdf

19-00015-Reiss-Supplement3.pdf

## Notes

**Peer Reviewed**

**Funding:** This study is funded by UK aid from the UK government. It was part-funded through the Strengthening Evidence for Programming on Unintended Pregnancy (STEP-UP) Research Consortium, managed by the Population Council. KR was funded by an ESRC PhD fellowship.

**Competing Interests:** KR, FT, TDN, SB, KF, KK, MD, YR, and KC were employed by Marie Stopes International or Marie Stopes Bangladesh and KA, EP, KB and JM by Ipas or Ipas Bangladesh as staff members and/or consultants while working on this study. Marie Stopes International, Marie Stopes Bangladesh, Ipas, and Ipas Bangladesh were responsible for the design and implementation of the intervention being evaluated. The other authors declare no conflict of interest.

## References

[1] National Institute of Population Research and Training (NIPORT), Mitra and Associates, ICF International. Bangladesh Demographic and Health Survey 2014. Dhaka, Bangladesh and Rockville, MD: NIPORT, Mitra and Associates, ICF International; 2016. http://dhsprogram.com/pubs/pdf/FR311/FR311.pdf. Accessed June 21, 2016.

[2] Sonfield A Hasstedt K Kavanaugh ML Anderson R. The Social and Economic Benefits of Women's Ability to Determine Whether and When to Have Children. New York, NY: Guttmacher Institute; 2013. https://www.guttmacher.org/sites/default/files/report_pdf/social-economic-benefits.pdf. Accessed June 21, 2019.

[3] Singh S Hossain A Maddow-Zimet I Vlassoff M Bhuiyan HU Ingerick M. The incidence of menstrual regulation procedures and abortion in Bangladesh, 2014. Int Perspect Sex Reprod Health. 2017;43(1):1–11. 10.1363/43e2417. 28930621

[4] World Health Organization Department of Reproductive Health and Research (WHO/RHR), Johns Hopkins Bloomberg School of Public Health/Center for Communication Programs (CCP), Knowledge for Health Project. Family Planning: A Global Handbook for Providers (2018 Update). Baltimore, MD, and Geneva, Switzerland: CCP and WHO; 2018. http://apps.who.int/iris/bitstream/handle/10665/260156/9780999203705-eng.pdf. Accessed June 21, 2019.

[5] Speidel JJ Harper CC Shields WC. The potential of long-acting reversible contraception to decrease unintended pregnancy. Contraception. 2008;78(3):197–200. 10.1016/j.contraception.2008.06.001. 18692608

[6] Peipert JF Zhao Q Allsworth JE. Continuation and satisfaction of reversible contraception. Obstet Gynecol. 2011;117(5):1105–1113. 10.1097/AOG.0b013e31821188ad. 21508749 PMC3548669

[7] Machiyama K Huda FA Ahmmed F. Women's attitudes and beliefs towards specific contraceptive methods in Bangladesh and Kenya. Reprod Health. 2018;15(1):75. 10.1186/s12978-018-0514-7. 29739429 PMC5941610

[8] Smith C Ngo TD Gold J. Effect of a mobile phone-based intervention on post-abortion contraception: a randomized controlled trial in Cambodia. Bull World Health Organ. 2015;93(12):842–850A. 10.2471/BLT.15.160267. 26668436 PMC4669734

[9] Government of the People's Republic of Bangladesh, Directorate General of Family Planning; Kingdom of the Netherlands; World Health Organization, Bangladesh Country Office. Bangladesh National Menstrual Regulation Service Guidelines. Dhaka, Bangladesh: the Directorate General; 2014. https://srhr.org/abortion-policies/documents/countries/05-BANGLADESH-NATIONAL-MENSTRUAL-REGULATION-SERVICES-GUIDELINES.pdf. Accessed June 21, 2019.

[10] Government of the People's Republic of Bangladesh, Directorate General of Family Planning, MCH-Services Unit. Memorandum No.: DGFP/MCH-RH/pro-Sha (Admin) 23/05/108. Dhaka, Bangladesh: the Directorate General; 2015.

[11] Sultana F Nahar Q Marions L Oliveras E. Effect of post-menstrual regulation family-planning service quality on subsequent contraceptive use in Bangladesh. Int J Gynaecol Obstet. 2013;123(suppl 1):e38–e42. 10.1016/j.ijgo.2013.07.007. 23992622

[12] Smith C Vannak U Sokhey L Ngo TD Gold J Free C. Mobile Technology for Improved Family Planning (MOTIF): the development of a mobile phone-based (mHealth) intervention to support post-abortion family planning (PAFP) in Cambodia. Reprod Health. 2015;13(1):1. 10.1186/s12978-015-0112-x. 26728505 PMC4700587

[13] Free C Phillips G Galli L. The effectiveness of mobile-health technology-based health behaviour change or disease management interventions for health care consumers: a systematic review. PLoS Med. 2013;10(1):e1001362. 10.1371/journal.pmed.1001362. 23349621 PMC3548655

[14] Free C McCarthy O French RS. Can text messages increase safer sex behaviours in young people? Intervention development and pilot randomised controlled trial. Health Technol Assess. 2016;20(57):1–82. 10.3310/hta20570. 27483185 PMC4983705

[15] French RS McCarthy O Baraitser P Wellings K Bailey JV Free C. Young people's views and experiences of a mobile phone texting intervention to promote safer sex behavior. JMIR Mhealth Uhealth. 2016;4(2):e26. 10.2196/mhealth.4302. 27083784 PMC4851722

[16] Smith C Gold J Ngo TD Sumpter C Free C. Mobile phone-based interventions for improving contraception use. Cochrane Database Syst Rev. 2015;(6):CD011159. 10.1002/14651858.CD011159.pub2. 26115146 PMC6485989

[17] Horvath T Azman H Kennedy GE Rutherford GW. Mobile phone text messaging for promoting adherence to antiretroviral therapy in patients with HIV infection. Cochrane Database Syst Rev. 2012;(3):CD009756. 10.1002/14651858.CD009756. 22419345 PMC6486190

[18] Daher J Vijh R Linthwaite B. Do digital innovations for HIV and sexually transmitted infections work? Results from a systematic review (1996-2017). BMJ Open. 2017;7(11):e017604. 10.1136/bmjopen-2017-017604. 29101138 PMC5695353

[19] Mimiaga MJ Thomas B Biello K. A pilot randomized controlled trial of an integrated in-person and mobile phone delivered counseling and text messaging intervention to reduce HIV transmission risk among male sex workers in Chennai, India. AIDS Behav. 2017;21(11):3172–3181. 10.1007/s10461-017-1884-5. 28831618 PMC5784829

[20] Constant D de Tolly K Harries J Myer L. Mobile phone messages to provide support to women during the home phase of medical abortion in South Africa: a randomised controlled trial. Contraception. 2014;90(3):226–233. 10.1016/j.contraception.2014.04.009. 24850188

[21] Patel SJ Subbiah S Jones R. Providing support to pregnant women and new mothers through moderated WhatsApp groups: a feasibility study. mHealth. 2018;4:14. 10.21037/mhealth.2018.04.05. 29963559 PMC5994467

[22] Mobile Alliance for Maternal Action (MAMA). Lessons from Country Programs Implementing the Mobile Alliance for Maternal Action Programs in Bangladesh, South Africa, India and Nigeria, 2010-2016. MAMA; 2017. https://www.mcsprogram.org/resource/mama-lessons-learned-report/. Accessed July 21, 2019.

[23] World Health Organization (WHO). Consolidated Guidelines on the Use of Antiretroviral Drugs for Treating and Preventing HIV Infection: Recommendations for a Public Health Approach. 2nd ed. Geneva, Switzerland: WHO; 2016. http://apps.who.int/iris/bitstream/10665/208825/1/9789241549684_eng.pdf. Accessed June 21, 2019. 27466667

[24] Sondaal SFV Browne JL Amoakoh-Coleman M. Assessing the effect of mHealth interventions in improving maternal and neonatal care in low- and middle-income countries: a systematic review. PLoS One. 2016;11(5):e0154664. 10.1371/journal.pone.0154664. 27144393 PMC4856298

[25] Johnson D Juras R Riley P. A randomized controlled trial of the impact of a family planning mHealth service on knowledge and use of contraception. Contraception. 2017;95(1):90–97. 10.1016/j.contraception.2016.07.009. 27421767

[26] Maslowsky J Frost S Hendrick CE Trujillo Cruz FO Merajver SD. Effects of postpartum mobile phone-based education on maternal and infant health in Ecuador. Int J Gynaecol Obstet. 2016;134(1):93–98. 10.1016/j.ijgo.2015.12.008. 27126905 PMC5927590

[27] Biswas KK Hossain A Chowdhury R. Using mHealth to support postabortion contraceptive use: results from a feasibility study in urban Bangladesh. JMIR Formative Research. 2017;1(1):e4. 10.2196/formative.5151. 30684398 PMC6334674

[28] Blanc AK. The effect of power in sexual relationships on sexual and reproductive health: an examination of the evidence. Stud Fam Plann. 2001;32(3):189–213. 10.1111/j.1728-4465.2001.00189.x. 11677692

[29] Bawah AA Akweongo P Simmons R Phillips JF. Women's fears and men's anxieties: the impact of family planning on gender relations in northern Ghana. Stud Fam Plann. 1999;30(1):54–66. 10.1111/j.1728-4465.1999.00054.x. 10216896

[30] Schuler SR Nazneen S. Does intimate partner violence decline as women's empowerment becomes normative? Perspectives of Bangladeshi women. World Dev. 2018;101:284–292. 10.1016/j.worlddev.2017.09.005. 29371749 PMC5777596

[31] Jennings L Gagliardi L. Influence of mHealth interventions on gender relations in developing countries: a systematic literature review. Int J Equity Health. 2013;12(1):85. 10.1186/1475-9276-12-85. 24131553 PMC4015705

[32] Bonell C Jamal F Melendez-Torres GJ Cummins S. ‘Dark logic’: theorising the harmful consequences of public health interventions. J Epidemiol Community Health. 2015;69(1):95–98. 10.1136/jech-2014-204671. 25403381

[33] Unger JA Ronen K Perrier T. Short message service communication improves exclusive breastfeeding and early postpartum contraception in a low- to middle-income country setting: a randomised trial. BJOG. 2018;125(12):1620–1629. 10.1111/1471-0528.15337. 29924912 PMC6179930

[34] McCarthy O Ahamed I Kulaeva F. A randomized controlled trial of an intervention delivered by mobile phone app instant messaging to increase the acceptability of effective contraception among young people in Tajikistan. Reprod Health. 2018;15(1):28. 10.1186/s12978-018-0473-z. 29433506 PMC5809875

[35] Trent M Thompson C Tomaszewski K. Text messaging support for urban adolescents and young adults using injectable contraception: outcomes of the DepoText pilot trial. J Adolesc Health. 2015;57(1):100–106. 10.1016/j.jadohealth.2015.03.008. 26002432 PMC4478161

[36] Castaño PM Bynum JY Andrés R Lara M Westhoff C. Effect of daily text messages on oral contraceptive continuation: a randomized controlled trial. Obstet Gynecol. 2012;119(1):14–20. 10.1097/AOG.0b013e31823d4167. 22143257

[37] Rokicki S Cohen J Salomon JA. Impact of a text-messaging program on adolescent reproductive health: a cluster-randomized trial in Ghana. Am J Public Health. 2017;107(2):298–306. 10.2105/AJPH.2016.303562. 27997236 PMC5227930

[38] Bull S Devine S Schmiege SJ Pickard L Campbell J Shlay JC. Text messaging, teen outreach program, and sexual health behavior: a cluster randomized trial. Am J Public Health. 2016;106(S1):S117–S124. 10.2105/AJPH.2016.303363. 27689478 PMC5049474

[39] Tsur L Kozer E Berkovitch M. The effect of drug consultation center guidance on contraceptive use among women using isotretinoin: a randomized, controlled study. J Womens Health (Larchmt). 2008;17(4):579–584. 10.1089/jwh.2007.0623. 18447762

[40] Harrington EK. Evaluation of an mHealth SMS Dialogue Strategy to Meet Women's and Couples' Postpartum Contraceptive Needs in Kenya (Mobile WACh XY): A Randomized Controlled Trial [master's thesis]. Seattle: University of Washington; 2017. https://digital.lib.washington.edu/researchworks/handle/1773/41691. Accessed June 21, 2019.

[41] Hou MY Hurwitz S Kavanagh E Fortin J Goldberg AB. Using daily text-message reminders to improve adherence with oral contraceptives: a randomized controlled trial. Obstet Gynecol. 2010;116(3):633–640. 10.1097/AOG.0b013e3181eb6b0f. 20733446

[42] Halpern V Lopez LM Grimes DA Stockton LL Gallo MF. Strategies to improve adherence and acceptability of hormonal methods of contraception. Cochrane Database Syst Rev. 2013;(10):CD004317. 10.1002/14651858.CD004317.pub4. 24163097

[43] Smith C Ly S Uk V Warnock R Edwards P Free C. Process evaluation of a mobile phone-based intervention to support post-abortion contraception in Cambodia. Contracept Reprod Med. 2017;2(1):16. 10.1186/s40834-017-0043-8. 29201421 PMC5683466

[44] Michie S Atkins L West R. The Behaviour Change Wheel: A Guide to Designing Interventions. Surrey, UK: Silverback Publishing; 2014.

[45] Bangladesh Bureau of Statistics. Violence Against Women (VAW) Survey 2015. Dhaka: Bangladesh Bureau of Statistics; 2016.

[46] Reiss K Andersen K Barnard S. Using automated voice messages linked to telephone counselling to increase post-menstrual regulation contraceptive uptake and continuation in Bangladesh: study protocol for a randomised controlled trial. BMC Public Health. 2017;17(1):769. 10.1186/s12889-017-4703-z. 28974209 PMC5627401

[47] Naved RT Samuels F Le Masson V Talukder A Gupta T Yount KM. Understanding Intimate Partner Violence in Rural Bangladesh: Prevention and Response. London, UK: Overseas Development Institute; 2017. https://www.odi.org/sites/odi.org.uk/files/resource-documents/11517.pdf. Accessed June 21, 2019.

[48] Innovations for Poverty Action. Poverty Probability Index. https://www.povertyindex.org/. Accessed June 21, 2019.

[49] Roberts C Torgerson DJ. Understanding controlled trials: baseline imbalance in randomised controlled trials. BMJ. 1999;319(7203):185. 10.1136/bmj.319.7203.185. 10406763 PMC1116277

[50] Carpenter JR Kenward MG. Multiple Imputation and Its Application. Chichester, UK: Wiley; 2013.

[51] Pallitto CC García-Moreno C Jansen HAFM Heise L Ellsberg M Watts C. Intimate partner violence, abortion, and unintended pregnancy: Results from the WHO Multi-country Study on Women's Health and Domestic Violence. Int J Gynecol Obstet. 2013;120(1):3–9. 10.1016/j.ijgo.2012.07.003 22959631

