# Supplementary material for: Unintended Consequences of mHealth Interactive Voice Messages Promoting Contraceptive Use After Menstrual Regulation in Bangladesh: Intimate Partner Violence Results From a Randomized Controlled Trial
Source: Glob Health Sci Pract. 2019 Sep 23;7(3):386–403. doi: 10.9745/GHSP-D-19-00015 (PMC6816818; doi:10.9745/GHSP-D-19-00015)
Supplement: 19-00015-Reiss-Supplement1.pdf [file 19-00015-Reiss-Supplement1.pdf]

Reiss K, Andersen K, Pearson E, et al. Unintended consequences of mHealth interactive voice messages promoting contraceptive use after menstrual regulation in Bangladesh: intimate partner violence results from a randomized controlled trial. Glob Health Sci Pract. 2019;7(3). https://doi.org/10.9745/GHSP-D-19-00015

SUPPLEMENT 1. Intervention Development and Description

Intervention Development

The intervention was adapted from a text message feasibility pilot conducted in Bangladesh[1] and drew on qualitative formative research,[2] behavior change theory,[3] and a review of existing literature on mHealth interventions for contraception and barriers and facilitators of contraceptive use in Bangladesh. During formative in-depth interviews, participants expressed a preference for voice messages over text and for content about the method of contraception they were using.[2] The intervention structure and draft content were developed during a workshop held in Bangladesh in June 2015 attended by individuals working for reproductive health NGOs in Bangladesh and internationally. Detailed content was developed in English by an international team and then adapted for the Bangladesh context and translated into Bangla during a 2-day workshop in Dhaka. Draft content was played to family planning clients at Marie Stopes International clinics and edits were made based on their feedback. Final content was reviewed by clinical teams in Chapel Hill and Dhaka, a family planning communications expert in the United States and the programs team at Marie Stopes International.

Message Timing and Cost to the User

The intervention comprised 11 automated interactive voice messages (more for switchers) sent to participant's mobile phones during their preferred time slot (8–10 a.m., 10 a.m.–3 p.m., 3–5 p.m., 5–9 p.m., or 9–11 p.m.), during the 4 months following recruitment. We aimed to send the first message out within 1 week of recruitment. The following 6 messages were sent at weekly intervals and the final 4 at fortnightly intervals. If a call was not delivered—for example, due to the phone being switched off or being out of network coverage or because the caller did not answer or was on another call—the call was attempted again after 30 minutes and again after another 30 minutes. After 3 attempts, no further call would be made until the next scheduled message. If the phone was answered, the automated content would start to play immediately. The messages and connecting calls to the call center were free to the participant.

Interactive Features

After the initial message was complete, participants were offered the following options: press 1 to repeat the initial message, press 2 to listen to more information about family planning, press 3 to speak to a call center counselor, press 4 to indicate that they were fine and did not need further information at that time, or press 5 to opt out from receiving further messages. Participants who selected option 2 were then asked to select to hear content about one of the following modern methods of contraception: condoms, pill, injection, implant, intrauterine device (IUD), male sterilization, female sterilization). After listening to the additional content, participants were returned to the first menu. Participants selecting to speak to a call center counsellor were diverted to 1 of 3 paramedics operating the study call center based at Marie Stopes Bangladesh head office. Participants selecting to opt out from receiving further messages were asked to confirm their choice before being disconnected. See the Figure on the next page.

Reiss K, Andersen K, Pearson E, et al. Unintended consequences of mHealth interactive voice messages promoting contraceptive use after menstrual regulation in Bangladesh: intimate partner violence results from a randomized controlled trial. Glob Health Sci Pract. 2019;7(3). https://doi.org/10.9745/GHSP-D-19-00015

FIGURE. Interactive Features of Each Voice Message

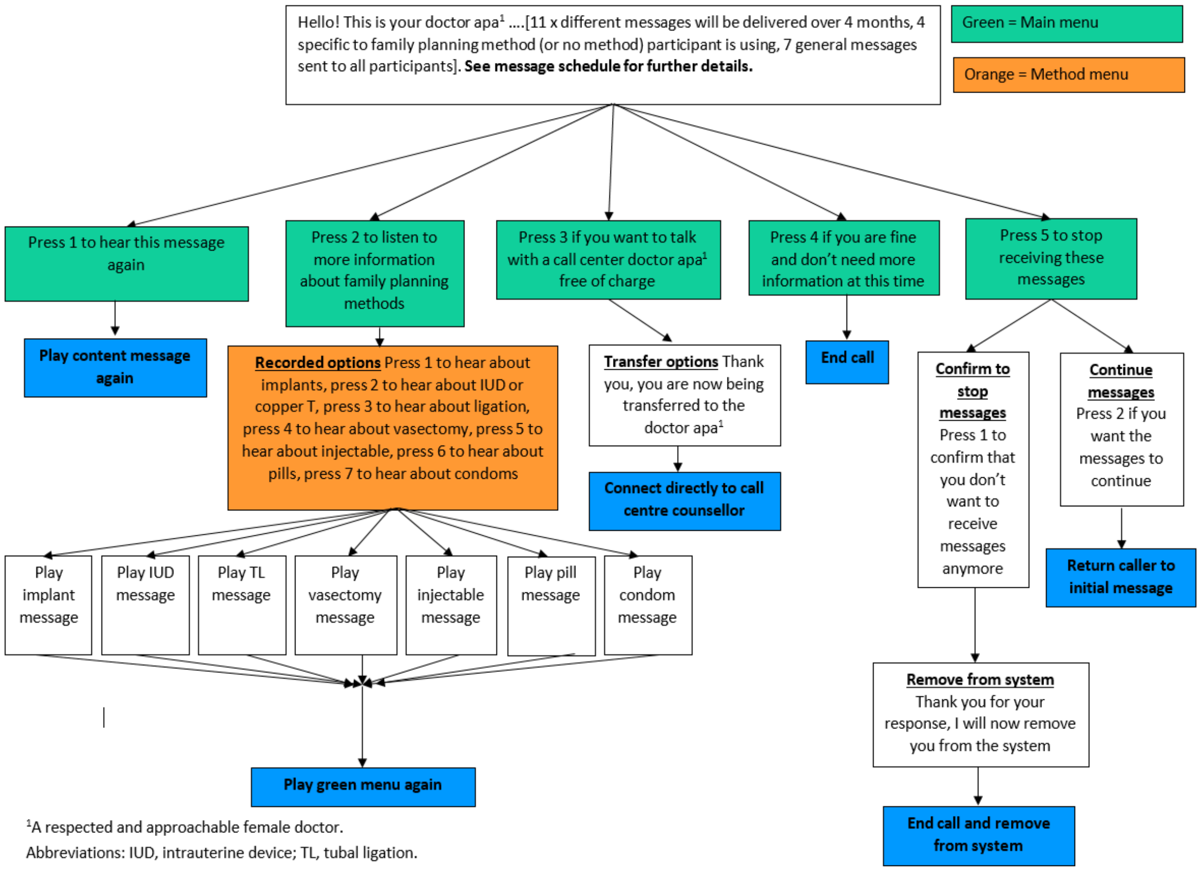

[1]A respected and approachable female doctor.

Abbreviations: IUD, intrauterine device; TL, tubal ligation.

Message Content and Content Tailoring

The initial automated content was different for each of the 11 messages and lasted for an average of 1 minute and 23 seconds. See Supplementary File 2 for full message content. The content aimed to increase motivation for using family planning, support continuation of the method of contraception the participant was using, address information gaps and misconceptions (particularly about LARCs and permanent methods), and encourage method switching for individuals who were not satisfied with their method. Messages also reminded participants to speak to a call center counselor if they needed further information or if they switched methods, and participants were given information on where to access family planning services. Eight messages were delivered by "doctor apa" a respected and approachable female doctor, 2 messages were "testimonials" in which a woman spoke of her experience using an implant or an IUD, and 1 was delivered by a female local celebrity (a well-known singer).

Participants were allocated to 1 of 6 intervention groups depending on the method of family planning they chose at the clinic after their menstrual regulation procedure (no method, condom, pill, injectable, implant or IUD). Their group allocation was updated if they reported switching methods to a counselor or research assistant. Four of the initial messages (those sent in weeks 2, 4, 6, and 10) were tailored to the family planning group. See the Table on the next page for the family

planning messages schedule. A participant's family planning group was changed if they reported switching methods to a counselor or research assistant, and the message series was restarted at message 2, the first method specific message, which meant that individuals who switched received more than 11 messages in total.

TABLE. Family Planning Message Schedule

No.	Weeks since put in system	Group 1<br>No method users	Group 2<br>Condom users	Group 3<br>Pill Users	Group 4<br>Injectable users	Group 5<br>Implant users	Group 6<br>IUD users
1.	0	**Generic Message 1a**<br>Welcome message no user	**Generic Message 1b**<br>Welcome message User	**Generic Message 1b**<br>Welcome message User	**Generic Message 1b**<br>Welcome message User	**Generic Message 1b**<br>Welcome message User	**Generic Message 1b**<br>Welcome message User
2.	1	**No method 1**<br>Reminder of benefit of contraception and availability of different methods	**Condom 1**<br>Increase motivation for and encourage trialing of condoms	**Pill 1**<br>Supporting correct use	**Injectable 1**<br>Correct use – return at 3 months	**Implant 1**<br>Motivation and reassurance around bleeding and return to fertility	**IUD 1**<br>Motivation – reminder of benefits and address fears around infertility
3.	2	**Generic Message 2**<br>IUD testimonial	**Generic Message 2**<br>IUD testimonial	**Generic Message 2**<br>IUD testimonial	**Generic Message 2**<br>IUD testimonial	**Generic Message 2**<br>IUD testimonial	**Generic Message 2**<br>IUD testimonial
4.	3	**No method 2**<br>Where to access contraception	**Condom 2**<br>Ensure correct use	**Pill 2**<br>Overcoming side effects/reminder of benefits	**Injectable 2**<br>Side effect management – irregular bleeding	**Implant 2**<br>Motivation – reminder of benefits	**IUD 2**<br>Support with side effects – bleeding changes. Reassure about return to fertility
5.	4	**Generic Message 3**<br>Implant testimonial	**Generic Message 3**<br>Implant testimonial	**Generic Message 3**<br>Implant testimonial	**Generic Message 3**<br>Implant testimonial	**Generic Message 3**<br>Implant testimonial	**Generic Message 3**<br>Implant testimonial
6.	5	**No method 3**<br>Fears around FP – side effects	**Condom 3**<br>Encourage switching if not happy with condoms	**Pill 3**<br>Addressing myths around taking breaks/infertility	**Injectable 3**<br>Return to fertility, no need to have a break	**Implant 3**<br>Motivation – remind benefits and support with bleeding	**IUD 3**<br>Motivation: Reinforce benefits Check implant place
7.	6	**Generic Message 4**<br>Return to fertility	**Generic Message 4**<br>Return to fertility	**Generic Message 4**<br>Return to fertility	**Generic Message 4**<br>Return to fertility	**Generic Message 4**<br>Return to fertility	**Generic Message 4**<br>Return to fertility
8.	8	**Generic Message 5**<br>Spouse / family communication	**Generic Message 5**<br>Spouse / family communication	**Generic Message 5**<br>Spouse / family communication	**Generic Message 5**<br>Spouse / family communication	**Generic Message 5**<br>Spouse / family communication	**Generic Message 5**<br>Spouse / family communication
9.	10	**Generic Message 6**<br>Sterilisation	**Generic Message 6**<br>Sterilisation	**Generic Message 6**<br>Sterilisation	**Generic Message 6**<br>Sterilisation	**Generic Message 6**<br>Sterilisation	**Generic Message 6**<br>Sterilisation
10.	12	**No method 4**<br>Switching method	**Condom 4**<br>Obtain support from husband / partner	**Pill 4**<br>Switching method	**Injectable 4**<br>Switching method	**Implant 4**<br>Advocacy message	**IUD 4**<br>Advocacy message
11.	14	**Generic Message 7**<br>Advocacy (recorded by celebrity)	**Generic Message 7**<br>Advocacy (recorded by celebrity)	**Generic Message 7**<br>Advocacy (recorded by celebrity)	**Generic Message 7**<br>Advocacy (recorded by celebrity)	**Generic Message 7**<br>Advocacy (recorded by celebrity)	**Generic Message 7**<br>Advocacy (recorded by celebrity)

Bibliography

1. Kanti Biswas K, Hossain A, Chowdhury R, et al. Using mHealth to support postabortion contraceptive use: results from a feasibility study in urban Bangladesh. *JMIR Form Res*. 2017;1(1):e4. doi:10.2196/formative.5151.

2. Eckersberger E, Pearson E, Andersen K, et al. Developing mHealth messages to promote postmenstrual regulation contraceptive use in Bangladesh: participatory interview study. *JMIR mHealth uHealth*. 2017;5(12):e174. doi:10.2196/mhealth.6969.

3. Michie S, Atkins L, West R. *The Behaviour Change Wheel: A Guide to Designing Interventions*. Silverback Publishing; 2014. http://www.behaviourchangewheel.com/about-wheel.
