# Supplementary material for: Unintended Consequences of mHealth Interactive Voice Messages Promoting Contraceptive Use After Menstrual Regulation in Bangladesh: Intimate Partner Violence Results From a Randomized Controlled Trial
Source: Glob Health Sci Pract. 2019 Sep 23;7(3):386–403. doi: 10.9745/GHSP-D-19-00015 (PMC6816818; doi:10.9745/GHSP-D-19-00015)
Supplement: 19-00015-Reiss-Supplement4.pdf [file 19-00015-Reiss-Supplement4.pdf]

Reiss K, Andersen K, Pearson E, et al. Unintended consequences of mHealth interactive voice messages promoting contraceptive use after menstrual regulation in Bangladesh: intimate partner violence results from a randomized controlled trial. Glob Health Sci Pract. 2019;7(3). https://doi.org/10.9745/GHSP-D-19-00015

SUPPLEMENT 4. LARC Use at 4-Month Follow-Up in Different Subgroups After the Mobile Phone Intervention for Post-MR Contraception

	**Intervention Arm**	**Control Arm**		
	**No. Using LARC/Total No. Respondents**	**No. Using LARC/Total No. Respondents**	**Unadjusted OR of LARC Use at 4 Months (95% CI)**	**Adjusted OR of LARC Use at 4 Months (95% CI)[a,b]**
Age, years				
Under 25	10/112 (9%)	10/115 (9%)	1.03 (0.41–2.58)	0.89 (0.21–3.68)
≥25	38/276 (14%)	49/268 (18%)	0.71 (0.45–1.13)	1.16 (0.52–2.59)
Education				
Up to and including primary	16/118 (14%)	15/85 (18%)	0.73 (0.34–1.58)	0.67 (0.19–2.36)
Over primary	32/271 (12%)	44/298 (15%)	0.77 (0.47–1.26)	1.30 (0.56–3.03)
SES				
<50% percentile PPI score	26/188 (14%)	26/174 (15%)	0.91 (0.48–1.19)	0.72 (0.29–1.79)
≥50% percentile PPI score	22/201 (10%)	33/209 (16%)	0.66 (0.37–1.17)	1.41 (0.50–4.00)
MR procedure type				
MVA	42/289 (15%)	50/273 (18%)	0.76 (0.48–1.19)	1.39 (0.61–3.19)
Medical	6/100 (6%)	9/110 (8%)	0.72 (0.25–2.09)	0.51 (0.12–2.14)
Experienced physical IPV in the last year				
No	44/246 (13%)	51/328 (16%)	0.79 (0.51–1.22)	1.07 (0.52–2.19)
Yes	4/42 (10%)	8/53 (15%)	0.59 (0.17–2.12)	0.59 (0.02–13.99)
Who makes decision about whether participant uses contraception				
Self	8/65 (12%)	8/60 (13%)	0.91 (0.32–2.61)	2.97 (0.30–29.75)
Self and someone else	36/273 (13%)	46/276 (17%)	0.76 (0.47–1.22)	0.84 (0.37–1.90)
Someone else	4/51 (8%)	5/47 (11%)	0.71 (0.18–2.84)	0.81 (0.08–7.87)
Facility type[c]				
Government clinic supported by Ipas	25/190 (13%)	28/195 (14%)	0.90 (0.51–1.62)	2.35 (0.80–6.87)
Marie Stopes International clinic	23/199 (12%)	31/188 (16%)	0.62 (0.37–1.18)	0.50 (0.18–1.141)

Abbreviations: CI, confidence interval; IPV, intimate partner violence; LARC, long-acting reversible contraceptive; MR, menstrual regulation; MVA, manual vacuum aspiration; OR, odds ratio; PPI, Poverty Probability Index; SES, socioeconomic status.

[a] Adjusted for baseline LARC use, SES, and age except where these are subgroup categories.

[b] Total number varies due to missing data (see Table 1).

[c] Subgroup not prespecified in the published protocol.
