# Supplementary material for: Unintended Consequences of mHealth Interactive Voice Messages Promoting Contraceptive Use After Menstrual Regulation in Bangladesh: Intimate Partner Violence Results From a Randomized Controlled Trial
Source: Glob Health Sci Pract. 2019 Sep 23;7(3):386–403. doi: 10.9745/GHSP-D-19-00015 (PMC6816818; doi:10.9745/GHSP-D-19-00015)
Supplement: 19-00015-Reiss-Supplement5.pdf [file 19-00015-Reiss-Supplement5.pdf]

Reiss K, Andersen K, Pearson E, et al. Unintended consequences of mHealth interactive voice messages promoting contraceptive use after menstrual regulation in Bangladesh: intimate partner violence results from a randomized controlled trial. Glob Health Sci Pract. 2019;7(3). https://doi.org/10.9745/GHSP-D-19-00015

SUPPLEMENT 5. Physical IPV at 4-Month Follow-Up in Different Subgroups After the Mobile Phone Intervention for Post-MR Contraception

	Intervention Arm	Control Arm		
	**No. Experiencing Violence/Total No. of Respondents**	**No. Experiencing Violence/Total No. of Respondents**	**Unadjusted OR of Physical IPV at 4 Months (95% CI)**	**Adjusted OR of Physical IPV at 4 Months (95% CI)[a]**
Age of participant, years				
<25	17/111 (15.3%)	11/115 (9.6%)	1.71 (0.76–3.84)	1.86 (0.74–4.68)
≥25	25/274 (9.1%)	14/267 (5.2%)	1.81 (0.92–3.57)	2.08 (1.02–4.26)
Age difference (participant and partner)				
<9 years	29/228 (12.7%)	19/230 (8.3%)	1.62 (0.88–2.98)	1.93 (0.97–3.83)
≥9 years	13/154 (8.4%)	6/149 (4.0%)	2.20 (0.81–5.94)	2.18 (0.79–6.07)
Education of participant				
Up to and including primary	16/117 (13.7%)	11/85 (12.9%)	1.07 (0.47–2.43)	1.52 (0.61–3.82)
Over primary	26/269 (9.7%)	14/297 (4.7%)	2.16 (1.10–4.23)	2.20 (1.07–4.54)
SES of participant				
<50 percentile PPI score	24/188 (12.8%)	19/174 (10.9%)	1.19 (0.63–2.27)	1.28 (0.64–2.56)
≥50 percentile PPI score	18/198 (9.1%)	6/208 (2.9%)	3.37 (1.31–8.67)	4.66 (1.69–12.86)
Husband occupation				
Not earning or low income	15/90 (16.7%)	12/73 (16.4%)	1.02 (0.44–2.33)	1.11 (0.42–2.90)
Student or higher income	26/294 (8.9%)	13/307 (4.2%)	2.19 (1.10–4.36)	2.52 (1.21–5.23)
Division				
Dhaka	22/234 (9.4%)	13/235 (5.5%)	1.77 (0.87–3.61)	2.25 (1.01–4.98)
Sylhet	5/48 (10.4%)	5/44 (11.4%)	0.91 (0.24–3.37)	0.58 (0.13–2.56)
Chittagong	15/104 (14.4%)	7/103 (6.8%)	2.31 (0.90–5.93)	2.81 (0.99–7.96)
Urban/rural location				

City	12/134 (9.0%)	6/154 (3.9%)	2.42 (0.88–6.65)	2.70 (0.91–8.00)
Town	13/95 (13.7%)	6/85 (7.0%)	2.09 (0.76–5.76)	2.56 (0.85–7.71)
Village	17/157 (10.8%)	13/142 (9.2%)	1.20 (0.56–2.58)	1.20 (0.51–2.83)
Parity				
0 or 1 child	16/133 (12.0%)	9/150 (6.0%)	2.14 (0.91–5.02)	2.17 (0.84–5.63)
2 or more children	26/253 (10.3%)	16/231 (6.9%)	1.54 (0.80–2.95)	1.85 (0.92–3.72)
Have used contraceptive before				
Yes	35/346 (10.1%)	23/345 (6.7%)	1.58 (0.91–2.73)	1.84 (1.01–3.36)
No	7/39 (18.0%)	2/34 (5.9%)	3.50 (0.67–18.15)	2.59 (0.44–15.2)
Have used MR before				
Yes	11/125 (8.8%)	10/113 (8.9%)	0.99 (0.41–2.44)	1.53 (0.56–4.17)
No	31/260 (11.9%)	15/269 (5.6%)	2.29 (1.21–4.36)	2.37 (1.18–4.75)
Has discussed family planning with husband				
Never or once	7/56 (12.5%)	2/53 (3.8%)	3.64 (0.72–18.4)	3.30 (0.22–48.75)
More than once	34/326 (10.4%)	23/327 (7.0%)	1.54 (0.89–2.68)	1.81 (1.00–3.28)
Involved in decisions about using contraception				
Yes	35/335 (10.5%)	22/336 (6.6%)	1.67 (0.95–2.90)	1.84 (1.01–3.36)
No	7/51 (13.7%)	3/46 (6.5%)	2.28 (0.55–9.40)	3.01 (0.53–17.05)
Husband *not* happy for her to use contraception				
Agree or strongly agree	9/110 (8.2%)	7/110 (6.4%)	1.31 (0.47–3.65)	1.36 (0.44–4.22)
Neither agree nor disagree, disagree, or strongly disagree	28/250 (11.2%)	13/245 (5.3%)	2.25 (1.14–4.46)	2.69 (1.28–5.65)
Husband knows about MR				
Yes	5/34 (14.7%)	4/27 (14.8%)	0.99 (0.24–4.12)	0.98 (0.17–5.75)
No	37/352 (10.5%)	21/354 (5.9%)	1.86 (1.07–3.25)	2.10 (1.16–3.83)
Right before participant became pregnant, husband wanted pregnancy				

Reiss K, Andersen K, Pearson E, et al. Unintended consequences of mHealth interactive voice messages promoting contraceptive use after menstrual regulation in Bangladesh: intimate partner violence results from a randomized controlled trial. Glob Health Sci Pract. 2019;7(3). https://doi.org/10.9745/GHSP-D-19-00015

Now or later	20/157 (12.7%)	9/167 (5.4%)	2.56 (1.13–5.81)	2.57 (1.07–6.17)
Never	15/184 (8.2%)	14/174 (8.1%)	1.01 (0.47–2.17)	1.14 (0.50–2.59)
Husband knows about study participation at baseline				
Yes	13/165 (7.9%)	10/168 (6.0%)	1.35 (0.58–3.17)	1.52 (0.59–3.91)
No	28/215 (13.2%)	15/204 (7.4%)	1.89 (0.98–3.65)	2.13 (1.05–4.34)
Reported experience of physical IPV in last year at baseline				
Yes	20/42 (47.6%)	11/53 (20.8%)	3.47 (1.41–8.52)	3.22 (1.27–8.16)
No	22/343 (6.4%)	14/327 (4.3%)	1.53 (0.77–3.05)	1.46 (0.73–2.92)
Shares phone with husband				
Yes	18/163 (11.0%)	9/138 (6.5%)	1.78 (0.77–4.10)	1.61 (0.69–3.77)
No	24/223 (10.8%)	16/244 (6.6%)	1.72 (0.89–3.33)	1.65 (0.85–3.21)

Abbreviations: CI, confidence interval; IPV, intimate partner violence; MR, menstrual regulation; OR, odds ratio; PPI, Poverty Probability Index; SES, socioeconomic status.

[a]Adjusted for SES (PPI), age of participant, and baseline experience of physical IPV in the past year except where these are explanatory variables.
