# Supplementary material for: Unintended Consequences of mHealth Interactive Voice Messages Promoting Contraceptive Use After Menstrual Regulation in Bangladesh: Intimate Partner Violence Results From a Randomized Controlled Trial
Source: Glob Health Sci Pract. 2019 Sep 23;7(3):386–403. doi: 10.9745/GHSP-D-19-00015 (PMC6816818; doi:10.9745/GHSP-D-19-00015)
Supplement: 19-00015-Reiss-Supplement2.pdf [file 19-00015-Reiss-Supplement2.pdf]

Reiss K, Andersen K, Pearson E, et al. Unintended consequences of mHealth interactive voice messages promoting contraceptive use after menstrual regulation in Bangladesh: intimate partner violence results from a randomized controlled trial. Glob Health Sci Pract. 2019;7(3). https://doi.org/10.9745/GHSP-D-19-00015

SUPPLEMENT 2. Content of All mHealth Messages

The Bangla version of the messages is available on request to the authors.

Wording at the end of each message:
To listen to the message again, press 1. To learn more about family planning methods, press 2. Press 3 to talk to a counselor. If you don't need any information now press 4. Press 5 to stop receiving these messages.

Generic Messages (sent to all intervention participants)

Generic Message 1a: Greeting/Welcome Message for Contraceptive User Groups
Hello, this is your doctor apa [friendly and trustworthy female doctor, literal translation is "doctor sister"] calling to congratulate you on your decision to use family planning and for using these messages. Over the next few months we will be checking in with you to ensure you get all the support you need so that you can stay focused on yourself and your family, without worries about an unplanned pregnancy. We are available to help you to use the method that suits your body and your life. If you have any questions about your method or other methods remember we are here to help. Our call center operators provide a confidential service and they can also speak to your husband or other family members.

Generic Message 1b: Greeting/Welcome Message for Nonusers
Hello, this is your doctor apa calling, many thanks for listening. I congratulate you for taking time to think about the health of yourself and your family. I want to let you know that if you adopt family planning, you won't have to worry about an unplanned pregnancy. There are lots of good contraceptive methods available, so don't forget to visit a quality health provider soon who can help you find a safe and reliable method. Or you can speak to our call center operators who provide a confidential service. They can also speak to your husband or other family members.

Generic Message 2: Intrauterine Device (IUD) Testimonial
Hello, my name is Selina and I'm calling as part of your doctor apa program to tell you about my experience with using an IUD. I have two beautiful children and last year my husband and I didn't know if we wanted to have any more, but we weren't ready to do sterilization. I visited my local clinic and they told me the IUD would help me avoid pregnancy for up to 10 years without having to remember anything and it's really safe! But I was nervous about having something inserted into my womb. In the end I decided to try it and was surprised at how quickly they put it in. I hardly felt anything, though I did experience a little more bleeding than normal for the first few months. I know that if we change our minds and want another child, I can get it out any time and my fertility will return to normal. I'm so happy that I overcame my fears—now I can focus on my family and my future without stress. We can help you decide whether this method would be good for you.

Generic Message 3: Return to Fertility
Hello, this is your weekly doctor apa check-in. We can all agree that children are "flowers of our lives" and form an essential part of our lives. It is important each child comes when you are ready to welcome it and give it the best life possible. That's why we are giving you information about modern family planning methods like the implant, the IUD, the injectable, pills and condoms. These methods provide protection against pregnancy while you use them and they can be used for a short period or long term. After you stop using them your fertility returns to normal. So are you ready?

Generic Message 4: Spouse/Family Communication

Hello, this is your weekly doctor apa check-in. Having support from the husband and family can make it easier to use contraception. Have you discussed together the positive benefits of using a modern contraceptive method, like having more time to spend with your husband and family, more energy to work, more money saved for the current children to have the best opportunities possible? If your husband or family members don't understand things about the use of family planning, you can also encourage them to ask questions and you can let them know they can call our friendly professionals with questions at any time on the number you have been given.

Generic Message 5: Implant Testimonial

Hello, my name is Mithila, and I'm calling as part of your doctor apa program to tell you why I chose to use an implant. We have been married for three years, and we knew we wanted to have children, but we didn't want them straight away. I started using the pill, but I was not very good at remembering to take it every day, which made me worry about getting pregnant. I decided to visit a provider at a nearby health center, who told me that the implant would protect me for up to three years and I would be free from the worry of unintended pregnancy. I was nervous about having something placed under the skin of my arm, but the doctor showed me where it would be placed, where it will remain, and how it will protect me from pregnancy. The insertion was quick—it only took about five minutes, and then I didn't have to worry about it anymore. My bleeding got a bit lighter after the first few months. Last year, I decided to have my implant removed, and within three months I was pregnant. Now that our baby is born, I am preparing to get an implant again. If you're interested in more facts about the implant, speak to a service provider or visit the hospital.

Generic Message 6: Sterilization

Hello, this is your doctor apa speaking. I have information for those who have completed their family. If you do not want any more children, have you heard about sterilization? Sterilization for men is called vasectomy and for women is tubal ligation. Both are very effective ways to prevent pregnancy. They are quick and simple surgical procedures that are performed by doctors and won't affect your health. You can go home on the same day as the procedure. After sterilization the sex drive remains the same and for women the menstrual cycle remains unchanged. Many couples decide that one of them will have sterilization because then they are free from the worry of unwanted pregnancy and they don't have to remember to do anything. If you want more information we are here to help, or you can go to family planning clinic.

Generic Message 7: Advocacy Message (Delivered by a Well-Known Bangladeshi Woman)

Hello, this is Momtaz calling from your doctor apa program. I believe that women in our country are very dedicated to their family and to society. This drives them to make good decisions about when they are ready to have children and ensure they can offer them the best possible support. Family planning can help couples to plan their family. Some women choose to use highly effective methods like the IUD and implant, these methods stop women from worrying about unplanned pregnancy. There is no need to remember anything when you use these methods which makes them easy to use, this allows women to focus on their families. I hope you make the smart choice and choose the method that you need. Tell your friends about your experience of using family planning and encourage them to use reliable methods.

IUD Messages (sent to IUD users only)

IUD Users Message 1

Hello, this is your doctor apa calling to congratulate you on your choice of an IUD. The IUD is one of the most effective forms of contraception—more effective and easier to use than methods like pills or condoms. Now that you have your IUD fitted, you do not have to do anything else to prevent pregnancy for up to ten years! If you decide to have another child before then, you can get the IUD removed and your fertility will return to normal straightaway. The IUD cannot be felt by the man or the woman during sex, which means that you can be stress free.

IUD Users Message 2

Hello, this is your doctor apa calling to check in with you. I hope you are happy with your IUD; you made a smart choice to choose such an effective and safe way to save your fertility until you want it. Some women experience changes to their menstrual bleeding when they have an IUD inserted. Your bleeding may get heavier or last for longer, especially in the first six months and you might also experience light spotting between periods, just before menstruation is due. Don't worry, this is normal! In most cases these changes will settle down after the first two to three months. If you have any problems which you think may be linked to your IUD, remember, we're here to help. You can talk to one of our counselors to check that everything is okay. Press 1…

IUD Users Message 3

Hello, this is your doctor apa calling to check in with you. Congratulations, you've now been using your IUD for a few months!! It's fantastic! I hope that you are enjoying not having to worry about pregnancy. Some women particularly like the IUD because it doesn't contain hormones—this can make it feel more natural. If you want to check your IUD is still inserted, first clean your hand with soap, then gently feel inside your vagina to see if the string is there. Don't pull on the string though as this is how it is removed. If you have any concerns, remember we're here to help! If for any questions or concerns about your IUD, please speak to our friendly professionals for advice.

IUD Users Message 4

Hello, this is your doctor apa calling to check in with you. I hope that you are now comfortable and confident with your IUD. Some women have worries about using an IUD, simply because they don't know enough about it, or they are told things that are not true. For example, some people worry that the IUD string can be seen when you urinate but this isn't true. Now that you know what the IUD is really like, you can help other women by talking about your experiences. Can you think of a family member, friend, or neighbor who might be interested in learning more about the IUD?

Implant Messages (sent to implant users only)

Implant Users Message 1

Hello, this is your doctor apa calling. Firstly, let me congratulate you for choosing to have an implant inserted. The implant is excellent at preventing pregnancy for 3 years. When you are ready to have a child, just have it removed and your fertility will return to normal within a short time. Even if you use it for the full three years your fertility will return to normal soon after its removal. Sometimes the implant can change the timing or amount of your menstruation and it can stop completely, this is normal.

Implant Users Message 2

Hello, this is your doctor apa speaking, I hope you are well. I want to remind you of some of the reasons so many women like using the implant. Firstly it is one of the most effective method of

preventing pregnancy. This means you and your husband can be stress free when you have sex. Once it is in, you don't have to remember or do anything! If you decide you want to get pregnant, just have it removed and your normal fertility will return within a short time. Don't forget though, you must use condoms too if you want to get protection against STIs [sexually transmitted infections]/HIV.

Implant Users Message 3
Congratulations, you've now been using your implant for a few months! I hope that you are enjoying not having to worry about pregnancy. You made a smart choice choosing one of the most effective methods of contraception. You might find that your monthly bleeding has changed. It may be lighter and less frequent or it may be more. You may also have some bleeding in between your monthly menstruation. This is normal. Whether you use the implant for a long or short time, your normal period and fertility will return after you have it removed.

Implant Users Message 4
This is your doctor apa, I hope that you are now comfortable and confident with the implant. Some women have worries about using an implant, simply because they don't know enough about it. Now that you know what the implant is like, you can help other women by talking about your experience. You can tell them that it is safe for your body and because you don't have to think about pregnancy, it can help you to focus your energy on the things that are important to you, whether that is your family, your work, or your studies. Perhaps the implant would also help them to plan their families? Can you think of someone you could encourage to try it?

Injectable Messages (sent to injectable users only)

Injectable Users Message 1
Hello, this is your doctor apa calling. Congratulations for using the injection. The injection is very effective in preventing pregnancy if it is taken every three months, but don't forget you need to use condoms if you want protection from STIs and HIV. If you miss your injection due date, it is important to return for your next injection as soon as you can. Avoid having sex during this time or use a condom to prevent pregnancy until you are able to get your next shot.

Injectable Users Message 2
Hello, this is your doctor apa calling. I hope you are well! Your regular bleeding pattern is likely to change while using the injection but you don't need to worry, this is normal. In the first three months you may have more irregular or prolonged bleeding. Over time your monthly bleeding may stop or become infrequent or irregular. These changes are normal and are not bad for your body. Some women using the injection will gain or lose a small amount of weight, this is a normal too. Menstruation will return to normal after you stop using the injection but sometimes this can take several months.

Injectable Users Message 3
Hello, this is your doctor apa calling. I hope you are well. The injectable is a safe method that prevents pregnancy for three months. You can keep using the injectable again and again every three months with no problem, even if you use it for long periods of time it still won't cause any problem. If you decide that you want another child, your fertility returns after stopping the injectable but some women may have to wait several months for it to return. You can talk to us if you have questions about becoming pregnant after using the injectable.

Injectable Users Message 4
Hello, this is your doctor apa calling. I hope you are well! Congratulations, you've now been using injectables for a few months! I hope that you are enjoying not having to worry about pregnancy. If you don't think the injectable is suited to you, don't forget there are many other family planning methods you could try. If you don't want to return to the clinic every three months, you could have an implant or an IUD fitted. Both are excellent at preventing pregnancy, and if you use them you don't have to remember to do anything. If you want to have children you can get the method removed.

Pill Messages (sent to pill users only)

Pill Users Message 1
Hello, this is your doctor apa calling to congratulate you for choosing pills! Pills are effective in preventing pregnancy if taken at the same time every day. Remember that to work, pills must be taken every day whether or not you have sex. If you miss a pill, always take one missed pill immediately, then continue with the pills in the pack on your normal schedule. If you miss three or more pills in a row, you are at risk for pregnancy so use condoms for seven days and consider using emergency contraception. If you have questions or concerns about using pills correctly, we are available 24 hours a day to give advice.

Pill Users Message 2
Hello, this is your doctor apa calling. I hope you are well. The pill is good for protecting pregnancy but don't forget you need to use condoms if you want protection from STIs and HIV. Some women who use pills experience changes to the amount and timing of their menstrual bleeding. It can get lighter, more regular or sometimes more irregular. Don't worry, this is normal and it may return to normal after a few months. If you have questions or concerns about the pill or if you want information about other methods we are here to help.

Pill Users Message 3
Hello, this is your doctor apa calling. I hope you are well. One of the great things about pills is that you can take them for a short time, or for many, many years without causing any problems for your body. They give you the flexibility to save your fertility until the time is right for you. As soon as you decide that you want to become pregnant, you just stop taking the pills and your fertility will return to normal.

Pill Users Message 4
Hello, this is your doctor apa calling I hope you are well. Congratulations, you've now been using pills for a few months! That's fantastic, I hope that you are enjoying not having to worry about pregnancy. If you are not happy with using the pill or if you have trouble remembering to take it every day, don't forget there are other methods you can try which may be better suited to you. For example, the injectable, implant and IUD are excellent at preventing pregnancy until the right time and you don't have to remember to do something every day. Your fertility will return to normal shortly after you stop using them.

Condom Messages (sent to condom users only)

Condom Users Message 1
Hello again, this is your doctor apa calling to check in on you. Congratulations for choosing condoms, they are a good way to prevent pregnancy without having to taking any medicines and

they are the only method that protects against STIs and HIV. If you are not using condoms, why not try them out to see if they are suited to you? I have some ideas to help you get started. First, you could speak to your husband before sex about what family planning method you would like to use. You can tell him that many couples like condoms as they can help the man to last for longer, which gives more enjoyment. In addition to this it can reduce worry about getting pregnancy. If your husband thinks the condom won't suit him, you can tell him that there are different sizes that you can try. Secondly, it's a good idea to keep condoms somewhere where you can easily get them when you need them, like in the bedroom. Where do you think you can keep yours? Lastly, remember, if you have questions about condoms or if you want to learn about other ways to prevent pregnancy, we are here to help!

Condom Users Message 2
Hello again, this is your doctor apa, I hope you're doing fine. Condoms are a good way to prevent pregnancy if you use them every single time you have sex. They are easy to use correctly. I believe you can use them the right way. Let me remind you: It is essential that you or your partner puts the condom on before the penis and vagina touch. When the penis is erect, you need to carefully tear open the packet, then squeeze the top of the condom so no air is trapped. Next, roll it all the way to the bottom of the penis. After ejaculation, make sure you or your partner hold the condom on while you withdraw the penis from the vagina, so no semen spills. Now you can feel confident that you can prevent pregnancy. If you are worried about whether you are using it the right way, or if you want to learn about other methods of preventing pregnancy, we are here to help. We can even talk to your husband if he agrees.

Condom Users Message 3
Hello again from your doctor apa. I hope you're having a good week. I wanted to check if you feel that condoms are working okay for you. Are you using them correctly every time you have sexual intercourse? If they are not used properly, you could get pregnant. It's great to plan ahead to make sure you won't run out of condoms. If you are not sure whether the condom is suited to you or your husband, don't worry, there are some other fantastic ways to prevent pregnancy that are safe, very effective, and really easy to use. For example, many women like the IUD and implant because you don't have to remember anything. If you want to get pregnant again, you just have to visit the doctor and get these methods removed. This can save you time and stress.

Condom Users Message 4
This is your doctor apa calling. Does your husband know about the benefits of using a condom? You can remind him that they can allow you both to feel free from worry of pregnancy. They can help you feel closer as you are using a method together and can help lengthen the sexual intercourse. If your husband would like to know more, he can speak to our call center counselor anytime, using the number we gave you. If condoms don't suit you or your husband, don't forget that there are many other methods you could try. Some women choose condoms together with another method so they can feel extra secure against pregnancy, STIs, and HIV.

No Method Users (sent to nonusers only)

No Method Users Message 1

Hello, this is your doctor apa calling to check how you are. Modern contraceptives can let you and your husband feel stress free and enjoy your life because you know you are safe from becoming pregnant until you are ready. There are many different methods you can use; different types of contraceptives suit different women at different times of their lives. Let us help you to find one which best suited to your lifestyle and your body.

No Method Users Message 2

This is your doctor apa again. I hope you are fine! Are you ready to start a contraceptive method now? Why not give one a try? I have some tips for you on where to go. Marie Stopes and public sector clinics have a full range of methods available and they start by giving you counseling to help you to choose which is best suited to you. How would you feel to know that you are protected against pregnancy until the time is right? We can help if you want to know where to get these methods.

No Method Users Message 3

Hello, how are you doing? This is your doctor apa again. Many women around the world choose to use modern contraceptives because they are a safe and effective way to save your fertility until the time that is right for you. Most women who take them are side effect free; they can be so comfortable you can even forget that you are using something and can feel free to enjoy your life! If you start a method and you don't feel comfortable with it, you can always switch to something else. It's really easy to get started, I'm sure you can do it! Why don't you visit a clinic to find out which one is the best option for you? …

No method users Message 4

This is your doctor apa calling. I hope you are well! Different contraceptives suit different women at different times in their life. Which do you think would be the right one for you? Here are some advantages for you to think about: implants and IUDs are great for women who want to feel confident they are protected against pregnancy until the right time without having to remember to do something each day. Pills are good for women who want to know they will menstruate at the same time every month, condoms are the only option that will also protect against STIs and HIV, and sterilization is great for couples who have completed their family. What is most important to you? You don't have to decide alone.

Optional Messages (available at the end of each message by pressing button 2)

Optional Message: Tubal Ligation

Tubal ligation is a simple, quick, and safe surgery for women. It is also called female sterilization. It is a permanent method of birth control, and it cannot be reversed, and so it is for those women who do not want to have any more children. Many couples like this method because after doing this they are free from worry about getting pregnant. Women also like it because it has no negative effect on their health, it does not stop menstruation, and it has no effect on women's appearance or feelings. You can get it done at a clinic or hospital. In this procedure the fallopian tubes are blocked so that the egg cannot move through the tube and meet the sperm. It is quick and it becomes effective right

away. You can go home on the same day. Remember that if you want to know more about tubal ligation you can speak to our call center counselor. She can also speak with your husband or partner or other members of your family. Or, why not visit your nearest clinic for more information?

Optional Message: Vasectomy
Vasectomy is a simple and quick surgery for men. It is also called male sterilization. It is a permanent method of contraception, and it cannot be reversed, and so it is for those men who do not want children. The procedure is very simple. In vasectomy, the tube through which the sperm comes out is either cut or sealed. This prevents the sperm from meeting with the female egg. Vasectomy is a very safe and quick procedure. It takes about 30 minutes and starts working after about 3 months. You can use condoms during this time. Many couples like this procedure because afterwards that they are free from worry about getting pregnant. Couples also like it as it has no negative effect on health, sex drive, erections, and orgasms. It also means that women do not have to do or take anything for birth control. You can get it done from a clinic or hospital. If you want to know more about vasectomy, then you can speak with our call center counselor. She can also speak with your husband or partner or other members of your family. Or for more information why not visit your nearest clinic?

Optional Message: IUD
The IUD or Copper-T is placed in the womb and provides contraceptive protection for up to ten years. In Bangladesh it is available for women who have one child or more. Many women like it because it's safe and it's one of the most effective methods of contraception—and it allows you to have sex without fear of pregnancy. It is also popular because it doesn't contain any hormones and when you choose to have it removed, fertility returns immediately. Insertion of an IUD is a simple procedure that is done by a skilled provider and takes about 5 minutes. You can get an IUD from a health complex. It may increase monthly bleeding and cramps at first, but usually this settles down. If you have any more questions about it you can talk to our counselor.

Optional Message: Implant
The implant is one or two small plastic rods that sit under the skin of the inside of your upper arm. It slowly releases hormones and is excellent at preventing pregnancy, leaving you free to enjoy your life without having to remember anything. It only takes about 5 minutes to be inserted by a trained provider and then you can have sex without worrying about pregnancy. When you are ready to conceive, just return to the clinic to have it removed with the help of a doctor, and your fertility will go back to normal within a month. The implant is great for newly married women and for women with children because it allows you to focus on your life, knowing that you are saving your fertility for the right time.

Optional Message: Injectable
The injection is a great way to prevent pregnancy for three months. The injection only takes a few minutes and is usually given in the arm, thigh, or buttock by a trained provider. It can take up to seven days before the injection starts to work. You can use a condom for those 7 days. You can keep using the injectable again and again every three months with no problem. If you decide that you want another child, your fertility returns after stopping the injectable, but some women may have to wait several months for it to return.

Optional Message: Pill

If taken regularly, the pill can prevent the worry of experiencing unintended pregnancy. For the pill to work, it needs to be taken at the same time each day for three weeks and then you have a week's break. Some pills have slightly different daily requirements. You can take the pill for a short time, or for many, many years without causing any problems for your body. As soon as you decide that you want to become pregnant, you just stop taking the pills and your fertility will return to normal.

Optional Message: Condom

The male condom is a very thin rubber or plastic sheath that goes over the penis. It acts as a barrier to stop the man's sperm entering into the woman during sex. Condoms can protect against pregnancy, HIV, and STIs. But remember, in order for them to work, you have to use them correctly every time you have sex. Now I will remind you about how to use them correctly—it's easy to do once you know how, and I'm sure you can show your partner the right way. It is essential that you or your partner puts the condom on before the penis and vagina touch. When the penis is erect, you need to carefully tear open the packet, then squeeze the top of the condom so no air is trapped. Next, roll it all the way to the bottom of the penis. After ejaculation, make sure you or your partner hold the condom on while you withdraw the penis from the vagina, so no semen spills. If you want more information about how to use condoms or if you want to learn more about other methods, we are here to help. We can also speak to your husband.
