# Supplementary material for: Unintended Consequences of mHealth Interactive Voice Messages Promoting Contraceptive Use After Menstrual Regulation in Bangladesh: Intimate Partner Violence Results From a Randomized Controlled Trial
Source: Glob Health Sci Pract. 2019 Sep 23;7(3):386–403. doi: 10.9745/GHSP-D-19-00015 (PMC6816818; doi:10.9745/GHSP-D-19-00015)

Reiss K, Andersen K, Pearson E, et al. Unintended consequences of mHealth interactive voice messages promoting contraceptive use after menstrual regulation in Bangladesh: intimate partner violence results from a randomized controlled trial. Glob Health Sci Pract. 2019;7(3). https://doi.org/10.9745/GHSP-D-19-00015

SUPPLEMENT 3. Kaplan Meier Failure Estimates Among LARC Users, Pill Users, and Injectable Users

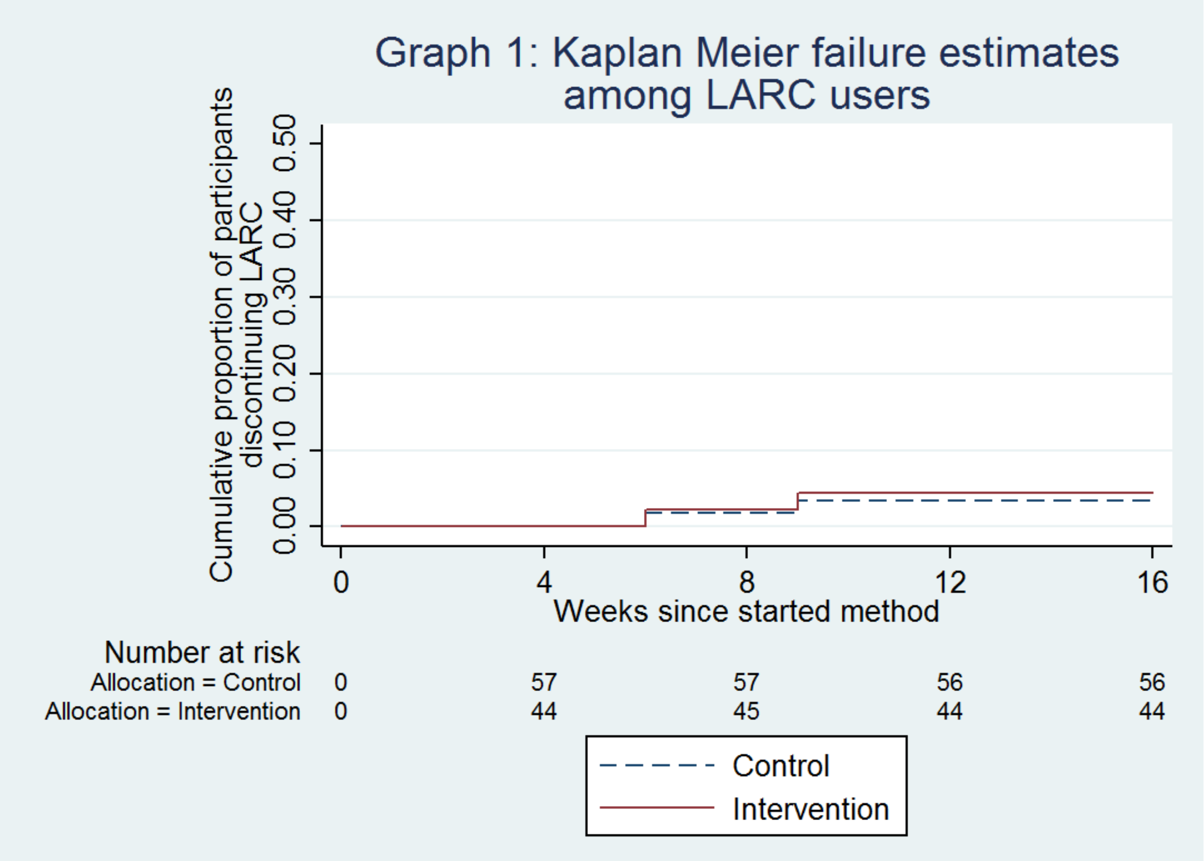

Abbreviation: LARC, long-acting contraceptive.

Reiss K, Andersen K, Pearson E, et al. Unintended consequences of mHealth interactive voice messages promoting contraceptive use after menstrual regulation in Bangladesh: intimate partner violence results from a randomized controlled trial. Glob Health Sci Pract. 2019;7(3). https://doi.org/10.9745/GHSP-D-19-00015

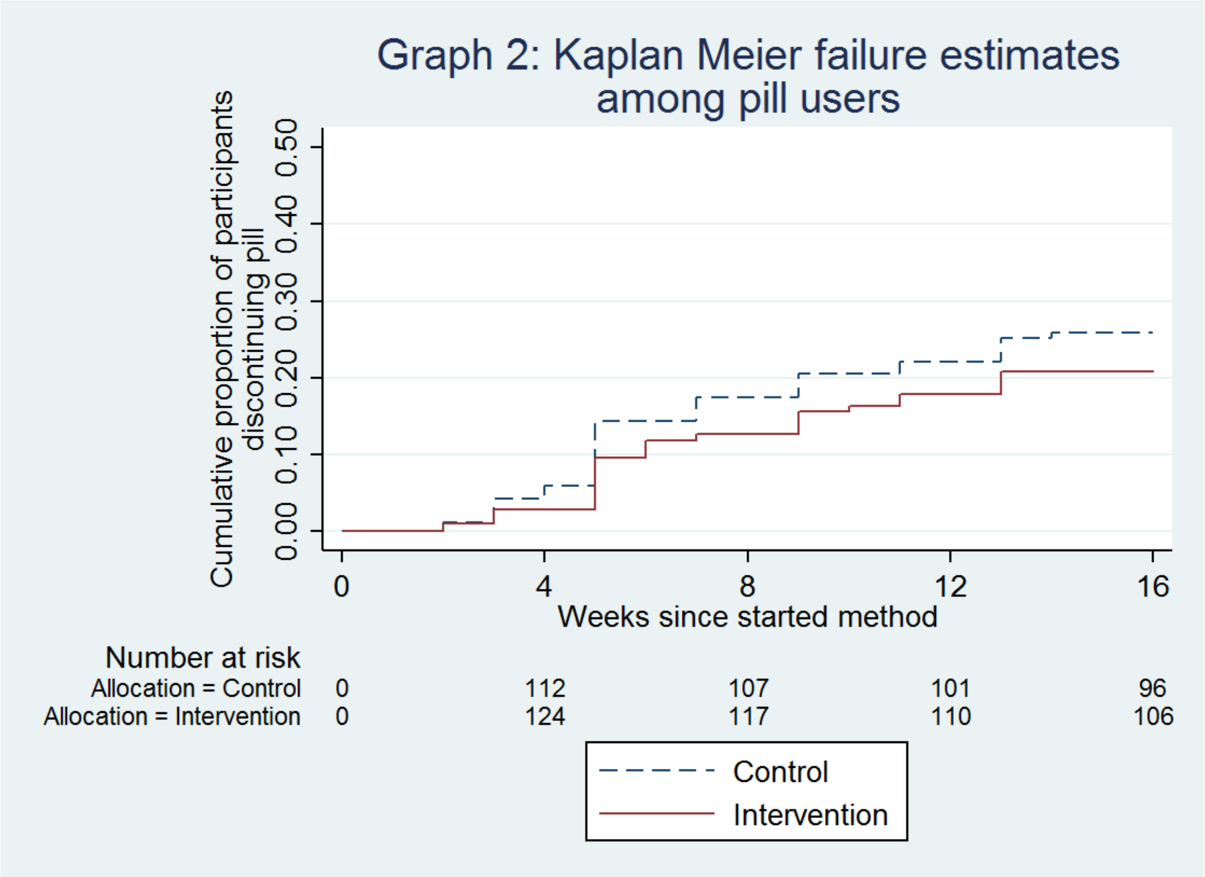

Reiss K, Andersen K, Pearson E, et al. Unintended consequences of mHealth interactive voice messages promoting contraceptive use after menstrual regulation in Bangladesh: intimate partner violence results from a randomized controlled trial. Glob Health Sci Pract. 2019;7(3). https://doi.org/10.9745/GHSP-D-19-00015

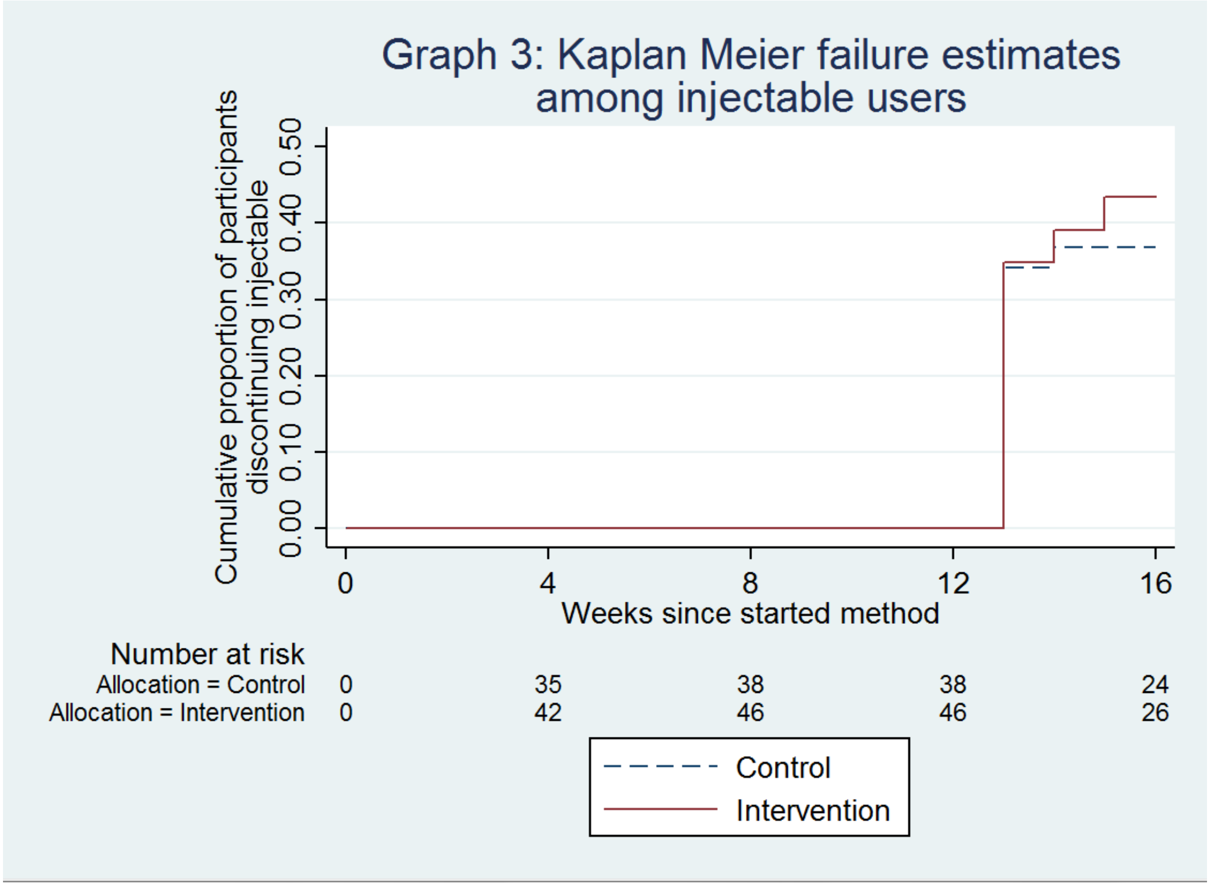

Supplement: 19-00015-Reiss-Supplement3.pdf [file 19-00015-Reiss-Supplement3.pdf]
